# Cardiovascular Concentration-Effect Relationships of Amodiaquine and its Metabolite Desethylamodiaquine: Clinical and Pre-clinical Studies

**DOI:** 10.1111/bcp.15569

**Published:** 2022-11-08

**Authors:** Xin Hui S Chan, Palang Chotsiri, Rebecca A Capel, James Pike, Borimas Hanboonkunupakarn, Sue J Lee, Maryam Hanafiah, Yan Naung Win, Maegan Cremer, Jean-René Kiechel, Bernhards Ogutu, Walter RJ Taylor, Rebecca-Ann B Burton, Joel Tarning, Nicholas J White

**Affiliations:** 1 Mahidol-Oxford Tropical Medicine Research Unit, Faculty of Tropical Medicine, Mahidol University, Bangkok, Thailand; 2 Centre for Tropical Medicine and Global Health, Nuffield Department of Medicine, University of Oxford, Oxford, United Kingdom; 3 Department of Pharmacology, University of Oxford, Oxford, UK; 4 Department of Clinical Tropical Medicine, Faculty of Tropical Medicine, Mahidol University, Bangkok, Thailand; 5 Department of Preventive and Social Medicine, University of Medicine, Taunggyi, Myanmar; 6 Drug for Neglected Diseases Initiative, Geneva, Switzerland; 7 Kenya Medical Research Institute, Kisumu, Kenya; 8 WorldWide Antimalarial Research Network, Centre for Tropical Medicine and Global Health, Nuffield Department of Medicine, University of Oxford, Oxford, United Kingdom

## Abstract

**Background:**

Amodiaquine is a 4-aminoquinoline antimalarial used extensively for the treatment and prevention of malaria. Orally administered amodiaquine is largely converted to the active metabolite desethylamodiaquine. Oral amodiaquine can cause bradycardia, hypotension, and electrocardiograph (ECG) QT interval prolongation in animals and humans but the relationship of these changes to drug concentrations is not well characterised.

**Methods:**

We conducted a secondary analysis of data from a pharmacokinetic study of the cardiac safety of amodiaquine (10mg/kg/day over 3 days) in 54 Kenyan adults (≥18 years) with uncomplicated malaria. Non-linear mixed effects modelling was used to assess amodiaquine and desethylamodiaquine concentration-effect relationships for vital sign (pulse rate, blood pressure) and ECG interval (QT, QRS, PR) outcomes. We also measured the spontaneous beating heart rate after cumulative dosing of amodiaquine and desethylamodiaquine in isolated mouse atrial preparations.

**Findings:**

Amodiaquine and desethylamodiaquine caused concentration-dependent mean decreases in pulse rate (1.9beats/minute per 100nmol/L), supine systolic blood pressure (1.7mmHg per 100nmol/L), erect systolic blood pressure (1.5mmHg per 100nmol/L), and erect diastolic blood pressure (1.4mmHg per 100nmol/L). The mean QT interval prolongation was 1.4milliseconds per 100nmol/L irrespective of correction factor used after adjustment for residual heart rate dependency. There was no significant independent effect of drug concentration on postural change in blood pressure or PR and QRS intervals. In mouse atria, the spontaneous beating rate was significantly reduced by amodiaquine (n=7) and desethylamodiaquine (n=8) at 3μmol/litre (amodiaquine: 10% ± 2%; desethylamodiaquine: 12% ± 3%) and 10μmol/litre (amodiaquine: 50% ± 7%; desethylamodiaquine: 46% ± 6%) concentrations with no significant difference in potency between the two compounds.

**Interpretation:**

Amodiaquine and desethylamodiaquine have concentration-dependent effects on heart rate, blood pressure, and ventricular repolarisation.

## Introduction

Amodiaquine (AQ), a 4-aminoquinoline structurally similar to chloroquine, is an important antimalarial drug which has been deployed extensively in the treatment and prevention of malaria over the past 70 years. First synthesised by the United States World War II (WWII) antimalarial research programme^1,2^, it was initially sold as monotherapy under the trade name of Camoquin® by the American pharmaceutical company Parke-Davis^2^. In 2005, the World Health Organization (WHO) recommended that artemisinin-based combination therapy (ACT) should become the first-line treatment for all falciparum malaria. In 2008, artesunate-amodiaquine (ASAQ), the fixed-dose formulation of amodiaquine and artesunate (AS), became the first major development product of a public-private partnership, the Drugs for Neglected Diseases initiative (DNDi). ASAQ is now recommended by the WHO for the treatment of uncomplicated *Plasmodium falciparum* and *P. vivax* malaria^3^ and is the first-line oral antimalarial in more than 20 African countries^4^ where malaria is endemic. Since 2012, WHO has recommended use of seasonal malaria chemoprevention (SMC) for young children (aged 3-59 months) living in areas of seasonal high-intensity malaria transmission in the Sahel subregion of Africa. SMC comprises a single dose of sulfadoxine-pyrimethamine together with amodiaquine (SP + AQ) divided over three days monthly during the rainy season for up to four months annually. Millions of children are now protected with SMC every year^5^.

The cardiovascular effects of amodiaquine have been recognised from the earliest studies in animal models^1^. During its development, pulsus bigeminus was noted in anaesthetised dogs receiving high doses of parenteral amodiaquine^1^. Like chloroquine, amodiaquine exhibits anti-arrhythmic properties, terminating experimental atrial arrhythmias in both decentralised and innervated canine hearts^6^ but, unlike chloroquine, does not appear to protect against experimental ventricular arrhythmias^7^. Electrocardiograph QT interval prolongation^7^, bradycardia^8^, and hypotension^8^ have also been observed after parenteral amodiaquine administration to anaesthetised dogs and cats. We have reported recently that amodiaquine prolongs the QT interval less, but is more bradycardic and hypotensive than chloroquine at current standard oral malaria treatment doses when given to adolescents and adults^9^. The clinical significance of these cardiovascular effects with standard malaria treatment dosing is unclear^9^ although bradycardia and hypotension may contribute to the higher incidence of mild asthenia and asthenia-like reactions after amodiaquine compared to other antimalarials^10–12^. Direct multiple ion channel blockade of cardiac^13–17^ and vascular^18^ myocytes along with altered autonomic tone^19,20^ may both be relevant. There are few data on the cardiovascular pharmacology of amodiaquine and information on its main metabolite desethylamodiaquine is especially limited, as the metabolite desethylamodiaquine was only identified in the 1980s^21^. This was around the time reports of the fatal toxicity of amodiaquine in chemoprophylaxis^22–24^ led to its temporary withdrawal in 1990^25^ from the list of WHO-recommended antimalarials.

We conducted a secondary analysis of a clinical pharmacokinetic study of ASAQ in adult malaria patients focusing on electrocardiographic interval (RR, QT, QRS, and PR) and cardiovascular vital sign (pulse rate and blood pressure) outcomes. This analysis of the clinical study was complemented by laboratory assessment of the concentration-heart rate response in murine atrial preparations with intact sino-atrial node (SAN).

## Methods

### Clinical

#### Trial Design

This was an open-label randomised controlled trial conducted between 2007 and 2008 in the Chulaimbo Sub-district Hospital of Kisumu, Kenya. The trial compared the fixed-dose ASAQ combination with loose (i.e. non-fixed dose) AS + AQ in adult patients (aged 18 to 60 years inclusive) presenting with acute uncomplicated *P. falciparum* monoinfection with an asexual parasitaemia of >1,000 parasites/μL and either a history of fever or a measured temperature of ≥37.5°C in the preceding 24 hours. Pregnant or lactating women, those with significant known comorbidities (including severe malnutrition or splenectomy), individuals with an ECG abnormality requiring urgent treatment, or those who had taken an artemisinin derivative and/or sulfadoxine-pyrimethamine in the previous three or seven days respectively were excluded. Further clinical details are reported in full elsewhere^26,27^.

#### Drug Regimen

Patients received either two fixed-dose ASAQ tablets (100/270mg, Sanofi-Aventis, France) daily for a total dose over three days of 600mg of AS and 1620mg of AQ, or four tablets each of non-fixed dose AS (Arsumax® 50mg, Sanofi-Aventis, France) and AQ (Flavoquine® 153mg, Sanofi-Aventis, France) daily for a total dose over three days of 600mg of AS and 1836mg of AQ. No concomitant food was given. All treatments were directly observed. Patients who vomited a dose within 1 hour after drug administration were retreated. Exact dosing times were recorded and used in modelling.

#### Clinical, Parasitological & ECG Procedures

Medical and drug histories were taken and physical examinations performed at baseline (D0). Basic clinical assessments including measurement of vital signs (pulse rate, supine and erect blood pressure, and axillary temperature) were conducted and malaria thick blood film slides were prepared at D0, D1, D2, D3, D7, D14, D21, and D28. A blood film was considered negative if no asexual parasites were seen after examination of 1000 white blood cells. Patients were asked about adverse events of headache, weakness, anorexia, nausea, abdominal pain, itching, vomiting, diarrhoea, rhinitis, cough, vertigo, and rash at each clinical assessment. Standard 12-lead ECGs were performed on D0 (pre-dose, +2 and +4 hours), D2 (+2 and +4 hours), and D28. ECGs were assessed centrally by cardiologists for arrhythmias and measurement of ECG intervals (RR, QT, QRS, and PR).

#### Blood Sampling Procedures

Full blood counts were performed on D0 (pre-dose), D7, and D28. Blood samples for drug concentration measurements were taken from all patients at fixed time points on D0 (pre-dose), D7, D14, D21, and D28. Each patient also had additional samples taken at random combinations of the following time points on D0 (+0.25, +0.5, +1, +1.5, +2, and +4 hours) and D2 (+0.25, +0.5, +1, +1.5, +2, and +4 hours). Exact sample times were recorded and used in the pharmacokinetic modelling.

#### Pharmacokinetic Methods

Venous plasma samples were analysed using liquid chromatography-mass spectrometry (LC-MS/MS) methods. Amodiaquine, desethylamodiaquine, and the internal standard (AST-D4) were analysed by reversed-phase liquid chromatography (X Terra C18 MS -3.5μm; 50mm x3mm id) and MS/MS (Sciex API3000) detection in the Turbo Ion Spray positive mode.

#### Ethics

The trial protocol was approved by the Kenya Medical Research Institute (KEMRI) Ethical Review Committee. Additional ethical approval for this secondary analysis of fully anonymised individual patient data was not deemed necessary in keeping with University of Oxford Central University Research Ethics Committee guidance.

#### Data Analysis

Trial data were standardised and checked according to a pre-specified data dictionary (Supplementary Appendix: Data Standardisation & Data Integrity Checks) for analysis. Measurements from fixed and non-fixed dose ASAQ arms were pooled as these dose formulations are known to be bioequivalent for amodiaquine and to not have an effect on the pharmacokinetic parameters of these drugs^27,28^. It is generally accepted that artesunate does not have a significant effect on the QT interval^29^.

In view of the inverse relationship between the QT interval and heart rate, measured QT intervals were adjusted for heart rate with the widely-used Bazett $\left(QTcB=\frac{QT}{\sqrt{RR}}\right)$ and Fridericia $\left(QTcF=\frac{QT}{\sqrt[{3}]{RR}}\right)$ correction formulae. A study-specific correction formula $\left(QTcS=\frac{QT}{RR^{0.42}}\right)$ was also applied with the correction exponent derived from log-log linear regression (Supplementary Appendix: Data Analysis – Study-Specific Heart Rate Correction). The QT interval was analysed as adjusted with the study-specific (QTcS), Fridericia (QTcF), and Bazett (QTcB) heart rate correction formulae.

All statistical analyses and data visualisation were done in R^30^ version 3.6.0, with linear mixed effects modelling conducted using the *nlme*^31^ package. Model fit was assessed by visual inspection of residuals while model discrimination was on the basis of likelihood ratio tests with *p* <0.05 as the threshold for statistical significance.

#### Pharmacokinetic Analysis

Observed amodiaquine and desethylamodiaquine concentrations, transformed into their natural logarithms, were analysed using non-linear mixed-effects modelling implemented in NONMEM version 7.4^32^ using the first-order conditional estimation with interactions method. Concentrations below the lower limit of quantification of 1ng/ml were omitted. In addition to R, Pirana^33^ version 2.9.0 and Perl-speaks-NONMEM (PSN)^34^ version 4.8.0 were used for automation, model evaluation, and diagnostics during the modelling process. The NONMEM $PRIOR functionality was used to stabilise model performance.

**Figure 1:**
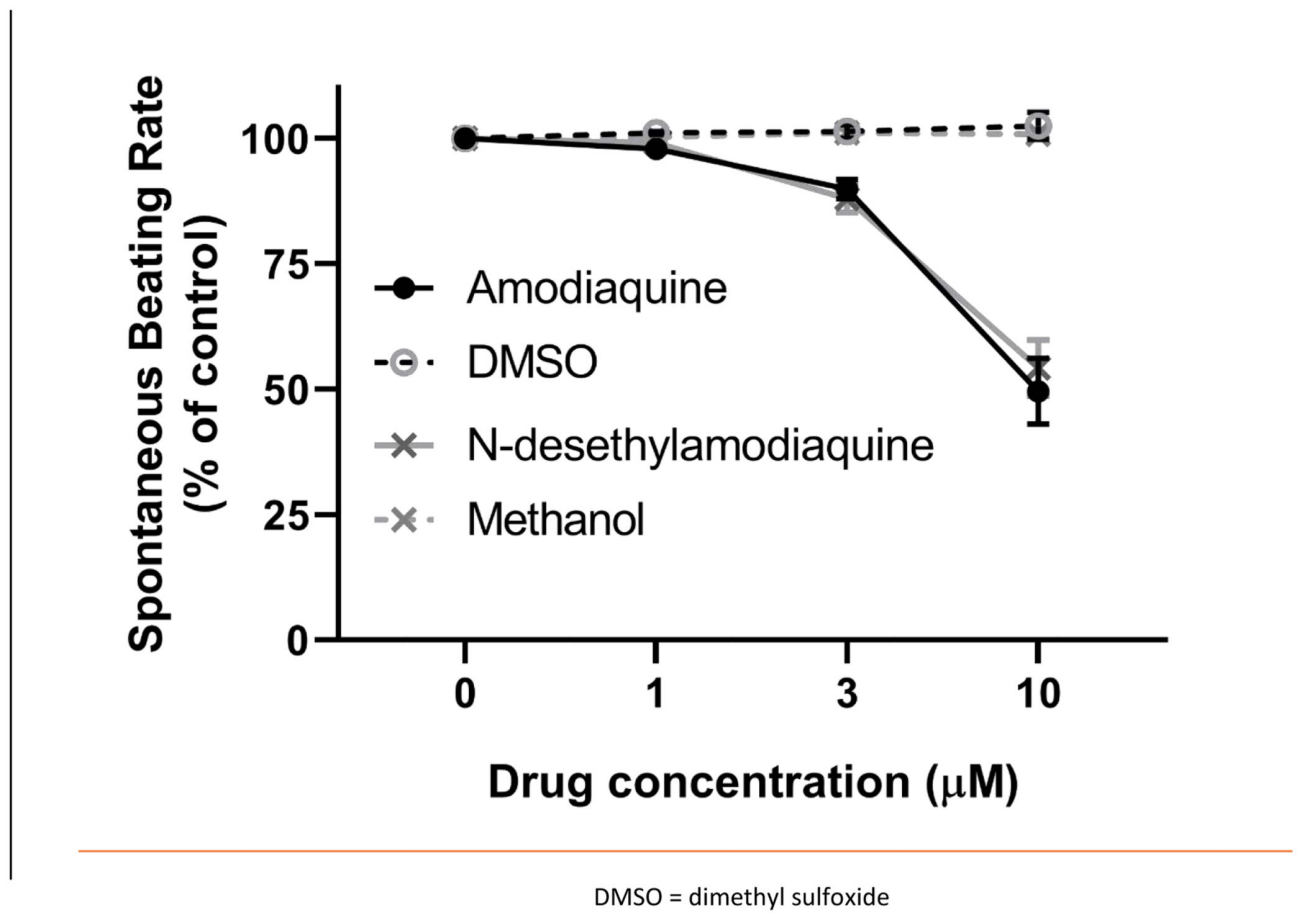
Change (%) in Atrial Beating Rate During Cumulative Doses of 4-aminoquinoline Antimalarials Compared to Controls

The structural pharmacokinetic model of amodiaquine and desethylamodiaquine was based on a model developed from another study of amodiaquine monotherapy for treatment of *P. vivax* malaria in pregnant women with rich (i.e. more intensive) pharmacokinetic sampling^35^ (Supplementary Appendix: Figure 1). Amodiaquine concentrations were described by lagged first-order absorption with a two-compartment distribution model followed by a three-compartment distribution model of desethylamodiaquine. Amodiaquine was assumed to be metabolised completely into desethylamodiaquine as the drug-metabolite conversion fraction was not identifiable. The final population pharmacokinetic parameter and inter-individual variability estimates with their parameter uncertainties from the previous study^35^ were incorporated into the model developed for this study as prior estimates (Supplementary Appendix: Data Analysis – Pharmacokinetic Analysis).

Predicted amodiaquine and desethylamodiaquine concentrations for the time points at which cardiovascular vital signs (pulse rate and blood pressure) and electrocardiographic intervals (RR, QT, QRS, and PR) were measured were used in the concentration-effect analyses.

#### Concentration-Effect Analyses

Multivariable linear mixed effects modelling was performed with the corrected QT, QRS, and PR intervals as well as the change from baseline of pulse rate and blood pressure (systolic and diastolic in the supine and erect positions with their postural differences) as response variables (Supplementary Appendix: Data Analysis – Concentration-Effect Analyses). Individual patient was the random effect while fixed effect selection was based on directed acyclic graphs of proposed causal relationships among available variables (Supplementary Appendix Figures II-V) identified from literature review^36^ and expert consultation^37^. The drug effect was evaluated using the total predicted concentration of amodiaquine plus its metabolite desethylamodiaquine based on prior evidence that both amodiaquine^6,7^ and desethylamodiaquine^38^ affect cardiovascular physiology as well as lack of evidence for any substantial difference in their activities from descriptive analyses. In the ECG interval models, the other fixed effects were body temperature change, age, and sex, with the addition of RR interval change to adjust for residual heart rate changes. For cardiovascular vital signs measured at multiple time points after recovery from malaria, the malaria disease effect was incorporated as a binary categorical fixed effect variable present during days 0-2 of treatment, with the addition of body temperature change and sex for the change in the pulse rate model only.

### Pre-clinical

#### Murine Atrial Studies

Adult male CD-1 mice (32-37 g, CD-1® IGS, Charles River Laboratories, UK) were housed maintained in a 12-hour light-dark cycle with *ad libitum* access to standard diet and sterilised water. Mice were culled by cervical dislocation in accordance with the United Kingdom Home Office Guidance on Animals (Scientific Procedures) Act 1986. The heart was excised rapidly and washed in heparin-containing Physiological Salt Solution (PSS, in millimoles: NaCl 125, NaHCO_3_ 25, KCl 5.4, NaH_2_PO_4_ 1.2, MgCl_2_ 1, glucose 5.5, CaCl_2_ 1.8, oxygenated with 95% O_2_/5% CO_2_). Ventricles were dissected away, the atria were separated at the atrial septum and the area adjacent to the SAN was cleared of connective tissue. The spontaneously-beating right atrial preparation was mounted in a 37°C organ bath containing PSS (continuously oxygenated with 95% O_2_/5% CO_2_) and connected to a force transducer (MLT0201 series, ADInstruments, New Zealand) with a resting tension of 0.2–0.3 g. The tension signal was low-pass filtered at 20Hz and the beating rate calculated from the time interval between contractions (LabChart, ADInstruments, New Zealand). After rate stabilisation in PSS (variation in average rate of a 10-second sample of no more than 2 beats/minute over a 10-minute period), cumulative concentrations of amodiaquine dihydrochloride (catalogue number 15314998, Acros Organics, Belgium) or N-desethylamodiaquine dihydrochloride solution (catalogue number D-039-1ML, Sigma Aldrich Co. Ltd., UK) were pipetted directly into the bath. Preparations were excluded if, under control conditions (PSS only), stabilised beating rate was less than 300 beats/minute or arrhythmic.

Data are presented as mean ± standard error of the mean (SEM). Repeated measures analysis of variance with Tukey’s or Dunnett’s correction as appropriate was used to assess the isolated heart findings.

## Results

### Clinical

54 patients aged between 18 and 60 years were randomised. Seven patients had received antimalarial pre-treatment: five patients chloroquine and two artemether-lumefantrine. None were receiving any cardiovascular concomitant medications. Baseline characteristics are presented in Table 1.

53 completed the full treatment course. One patient in the fixed-dose ASAQ arm withdrew consent. Four patients were subsequently lost to follow-up and censored in analyses at the time of drop-out. All patients recovered uneventfully. There were no serious cardiovascular events reported in any of the 54 patients.

#### Pharmacokinetic Analysis

A total of 363 post-dose venous plasma samples were collected from the 53 participants who received full treatment courses. Amodiaquine concentrations were measurable in 115 (31.7%) samples and 352 (97.0%) had measurable concentrations of desethylamodiaquine (Supplementary Appendix Figure VI).

Population pharmacokinetic parameter estimates were reliable, with small relative standard errors. Secondary pharmacokinetic parameters of maximum concentration, time to maximum concentration, terminal elimination half-life, and total exposure were also computed (Supplementary Appendix Table I). Goodness-of-fit diagnostics and the prediction-corrected visual predictive checks (Supplementary Appendix Figure VII-IX) demonstrated that the model described the observed data adequately.

#### Concentration-Effect Analyses – Cardiovascular Vital Signs

Plasma concentrations of amodiaquine and desethylamodiaquine were summed. The population mean maximum plasma total concentration (C_max_) of amodiaquine and desethylamodiaquine was approximately 750nmol/L (or 250ng/mL) (Supplementary Appendix Table I). This was associated with a mean decrease in pulse rate of 14.6 beats/minute (95% CI: 10.9 to 18.2). This effect was in addition to independent effects on pulse rate reduction following recovery from fever (5.7 beats/minute per 1°C decrease; 95% CI: 3.7 to 7.6) and from acute malaria (3.0 beats/minute; 95% CI: 0.5 to 5.5). Male sex was not associated with statistically significant effects on pulse rate compared to female sex at this sample size (Table 2).

After adjusting for acute malaria effects, the total plasma concentration of amodiaquine plus desethylamodiaquine at C_max_ was associated with a mean fall of 12.4mmHg (95% CI: 8.9 to 15.9) in supine systolic blood pressure and 11.0mmHg (95% CI: 7.4 to 14.7) in erect systolic blood pressure. Corresponding reductions in supine diastolic blood pressure were 4.7mmHg (95% CI: 1.9 to 7.4), and 10.3mmHg (95% CI: 7.4 to 13.1) in erect diastolic blood pressure (Table 2 & Supplementary Appendix Table III-IV). Total drug concentration effects on the postural change between supine and erect blood pressure measurements were small and of unclear significance (Supplementary Appendix Table III-IV).

#### Concentration-Effect Analyses – ECG Intervals

After adjustment for change in body temperature, age, sex, and change in RR interval, the mean corrected QT interval prolongation resulting from amodiaquine plus desethylamodiaquine at C_max_ was very similar irrespective of the heart rate correction factor used (QTcS: 10.4 milliseconds, 95% CI: 5.9 to 15.0; QTcF: 10.7 milliseconds, 95% CI: 6.1 to 15.2; QTcB: 10.3 milliseconds, 95% CI: 5.7 to 14.8). Unlike the study-specific corrected QT interval, the Fridericia- and Bazett-corrected QT intervals retained clinically and statistically significant heart rate dependency after adjustment although these were in opposite directions (QTcF: 11.6 milliseconds per 300-millisecond increase in RR interval, 95% CI: 7.3 to 16.0; QTcB: -11.5 milliseconds; 95% CI: -15.8 to -7.1). Thus, without adjustment, use of the Fridericia heart rate correction overestimated amodiaquine-related QT prolongation from baseline while the Bazett correction underestimated it (Table 3).

There was no significant effect of maximum plasma concentrations of amodiaquine plus desethylamodiaquine on the QRS and PR intervals once adjusted by change in body temperature, sex, age, and change in RR interval (QRS: -0.47 milliseconds, 95% CI: -2.51 to 1.57; PR: 2.01 milliseconds; 95% CI: -1.29 to 5.31) (Supplementary Appendix Table 5).

### Pre-Clinical

#### Murine Atrial Studies

Spontaneously beating right atrial preparations contain the intact SAN pacemaker. They can be used to assess the effect of compounds on the intrinsic pacemaker of the heart by measurement of beating rate^14^. The application of cumulative doses of amodiaquine (n=6) and N-desethylamodiaquine (n=8) to spontaneously beating mouse atrial preparations produced concentration-dependent reductions in beating rate which were statistically significant (*p* < 0.05) at concentrations of 3 μmol/litre (amodiaquine: 10% ± 2%; N-desethylamodiaquine: 12% ± 3%) and 10 μmol/litre (amodiaquine: 50% ± 7%; N-desethylamodiaquine: 46% ± 6%) but not 1 μmol/litre (amodiaquine: 2% ± 1%; N-desethylamodiaquine: 1% ± 1%) compared to time-matched controls of the vehicles dimethyl sulfoxide (n=2) and methanol (n=2). There was no statistically significant difference between the response to amodiaquine and N-desethylamodiaquine at any concentration studied (Figure 1).

## Discussion

The 4-aminoquinoline antimalarial amodiaquine is a pro-drug converted rapidly after oral administration to its active metabolite desethylamodiaquine by cytochrome P450 isozyme 2C8 (CYP2C8)^39^. Thus desethylamodiaquine, with its higher concentration-time profile and longer terminal elimination half-life of 9-18 days^40^, contributes almost all of the antimalarial effect of this widely-deployed oral drug in the treatment and prevention of malaria.

Despite its widespread use, amodiaquine has been relatively little studied in recent years. To our knowledge, this is the most detailed investigation to date of the cardiovascular concentration-effect relationships of amodiaquine and its active metabolite desethylamodiaquine, incorporating both clinical data in malaria patients and a pre-clinical study of the cardiac pharmacology of desethylamodiaquine.

### Cardiovascular Vital Signs

Bradycardia is a common cardiovascular effect after antimalarial treatment with amodiaquine and mefloquine^41^. It is observed more in adolescents and adults than in children^9,42^ for reasons which are not fully understood, although modulation of cardiac ion currents by sex hormones may play a role^43^.

Our murine atrial studies show that both amodiaquine and desethylamodiaquine have direct concentration-dependent bradycardic effects of similar potency. These effects are greater than that of hydroxychloroquine at the concentrations evaluated, measured using the same method in our previous study^14^. The amodiaquine-induced bradycardia in both innervated (malaria patients, anaesthetised dogs^8^) and decentralised (mouse) hearts supports a direct pharmacological effect on cardiac myocyte ion channels through modulation of the pacemaker If current at the SAN, as observed previously with hydroxychloroquine^14^. Autonomic tone may also be relevant as both amodiaquine and mefloquine are associated with reversible inhibition of human acetylcholinesterase (AChE), with amodiaquine having a much higher potency than mefloquine^19,20^.

The quinoline antimalarials quinine^44^ and chloroquine^45^ are known to cause lethal hypotension when injected rapidly but can be used safely with rate-controlled continuous intravenous infusion. As proposed for amodiaquine^18^, these hypotensive effects are likely due to vasodilation and negative inotropy from multiple ion channel blockade^46^. Orthostatic hypotension is a feature of acute malaria which is exacerbated by the quinoline antimalarials quinine and mefloquine^47^. However, in this study of uncomplicated malaria infections, there was no significant effect of the total plasma concentration of amodiaquine and desethylamodiaquine on postural changes in systolic or diastolic blood pressure after adjustment for malaria recovery.

Amodiaquine is the most bradycardic of the front-line antimalarials^9^. While amodiaquine and desethylamodiaquine cause concentration-dependent bradycardia and hypotension in adult malaria patients, the clinical impact of these effects appears to be mild^26^, although they may contribute to the commonly reported asthenia.

### ECG Intervals

Drug-induced QT interval prolongation is the most widely-used surrogate marker of the risk of development of torsades de pointes (TdP), a polymorphic ventricular tachycardia that can degenerate in some cases into ventricular fibrillation and cause sudden cardiac death^48^. Despite their QT-prolonging potential, the frontline quinoline and structurally-related antimalarials recommended currently by the WHO all have excellent track records of cardiac safety. They have not been associated with increased risk of sudden cardiac death or cases of TdP in their extensive use at standard doses for the treatment or prevention of malaria over seven decades^9,41,49^.

The QT interval lengthens as heart rate decreases. Correction formulae modelling this inverse and non-linear relationship are used to attempt to minimise the heart rate dependency of measured QT intervals to allow for comparisons across different heart rates. However, the commonly used Bazett and Fridericia corrections are both known to retain significant heart rate dependency^48,50^, particularly in malaria where there is further confounding from disease recovery which occurs as antimalarial concentrations peak^9,36^. As before^9^, a study-specific correction provided the best heart rate correction of the QT interval (QTcS) in our analysis while use of the Fridericia (QTcF) and Bazett (QTcB) corrections respectively overestimated and underestimated amodiaquine-related QT prolongation in malaria patients. Once any residual heart rate dependency had been adjusted for the drug-attributable QT prolongation from amodiaquine and desethylamodiaquine was comparable regardless of correction factor used and consistent with QT prolongation similar to that observed with piperaquine^51,52^ but less than with chloroquine^53^ at standard malaria doses.

In contrast, we found no significant effect of total amodiaquine and desethylamodiaquine concentration on PR or QRS intervals after adjustment for demographic factors (age, sex) and malaria recovery (change in body temperature, change in heart rate). Small increases in unadjusted PR and QRS intervals after amodiaquine for malaria have been previously reported^54,55^ which may predominantly reflect recovery from malaria rather than a direct drug effect. This differs from chloroquine^53^ which prolongs both the PR and QRS intervals with over one quarter of the QT prolongation following chloroquine resulting from QRS widening.

### Conclusion

The widely used 4-aminoquinoline amodiaquine, like other quinoline and quinoline-like drugs, has transient effects on cardiac and vascular physiology. We characterised the concentration-dependency of the bradycardic, hypotensive, and QT prolonging effects of amodiaquine and its main metabolite desethylamodiaquine providing further evidence of their causal role. Further characterisation of the cardiovascular effects of amodiaquine and desethylamodiaquine in adult healthy volunteers and other pre-clinical models may help to improve the tolerability of this important medicine in the treatment and prevention of malaria.

## Supplementary Material

Supplementary Appendix

## Figures and Tables

**Table 1:** Baseline Characteristics of Included Population (n = 54)

	
**Age (years)**	
Median (IQR)	24.0 (19.0-32.0)
18-<35	43 (79.6%)
35-<50	8 (14.8%)
≥50	3 (5.6%)
	
**Weight (kg)**	
Median (IQR)	59.0 (53.0-62.0)
	
**Sex**	
Female	29 (53.7%)
Male	25 (46.3%)
	
**Temperature (°C)**	
Median (IQR)	37.7 (37.1-38.6)
≥37.5	35 (64.8%)
	
**Parasitaemia (parasites/μL)**	
Median (IQR)	17181 (5317-25876)
>10,000-50,000	24 (44.4%)
>50,000-100,000	6 (11.1%)
>100,000-250,000	1 (1.9%)
	
**Haemoglobin (g/dL)**	
Median (IQR)	13.2 (11.9-14.8)
<8	0
	
**Heart Rate (beats/minute)**	
Mean (SD)	91.7 (15.7)^a^
≥140	0
120-<140	2 (3.8%)^a^
100-<120	17 (32.1%)^a^
80-<100	21 (39.6%)^a^
60-<80	13 (24.5%)^a^
<60	0

IQR = inter-quartile range, SD = standard deviation

a 1 participant had missing baseline heart rate

**Table 2:** Multivariable Linear Mixed Effects Regression Analysis of Pulse Rate and Blood Pressure in Malaria Following Treatment with Amodiaquine

		Pulse Rate (beats/minute)				Systolic Blood Pressure – Supine (mmHg)				Diastolic Blood Pressure – Erect (mmHg)			
		Univariable Analyses		Multivariable Analyses		Univariable Analyses		Multivariable Analyses		Univariable Analyses		Multivariable Analyses	
	Number of Observations	Crude Estimate (95% CI)	*p* value	Adjusted Estimate (95% CI)	*p* value	Crude Estimate (95% CI)	*p* value	Adjusted Estimate (95% CI)	*p* value	Crude Estimate (95% CI)	*p* value	Adjusted Estimate (95% CI)	*p* value
Total plasma concentration of amodiaquine and desethylamodiaquine, per 750^*^ nmol/litre increase	362	-19.51 (-23.10 to -15.92)	<0.0001	-14.57 (-18.22 to -10.92)	<0.0001	-18.09 (-20.99 to -15.20)	<0.0001	-12.43 (-15.95 to -8.92)	<0.0001	-10.64 (-13.03 to -8.25)	<0.0001	-10.26 (-13.11 to -7.42)	<0.0001
Body temperature change, per 1°C increase	362	9.72 (8.32 to 11.12)	<0.0001	5.65 (3.74 to 7.56)	<0.0001	4.46 (3.14 to 5.79)	<0.0001			2.97 (1.94 to 4.00)	<0.0001		
Malaria	362												
Yes (days 0-2)	106	-3.21 (-5.54 to -0.90)	0.0066	2.98 (0.50 to 5.46)	0.0186	-9.41 (-11.52 to -7.30)	<0.0001	-4.53 (-6.93 to -2.13)	0.0002	-1.13 (-2.84 to 0.58)	0.1958	2.91 (0.97 to 4.85)	0.0034
No (days 3-28)	256	Reference		Reference		Reference		Reference		Reference		Reference	
Sex	362												
Female	193	Reference		Reference		Reference				Reference			
Male	169	3.06 (-4.81 to 10.93)	0.4388	4.12 (-4.17 to 12.41)	0.3232	2.52 (-3.74 to 8.78)	0.4231			4.14 (-0.80 to 9.08)	0.0985		

* Mean maximum total plasma drug concentration (rounded) after a 3-day course of amodiaquine from pharmacokinetic analysis of same study

**Table 3:** Multivariable Linear Mixed Effects Regression Analysis of the Corrected QT Interval in Malaria Following Treatment with Amodiaquine

		QTcS – Study-specific Correction (milliseconds)				QTcF – Fridericia Correction (milliseconds)				QTcB – Bazett Correction (milliseconds)			
		Univariable Analyses		Multivariable Analyses		Univariable Analyses		Multivariable Analyses		Univariable Analyses		Multivariable Analyses	
	Number of Observations	Crude Estimate (95% CI)	*p* value	Adjusted Estimate (95% CI)	*p* value	Crude Estimate (95% CI)	*p* value	Adjusted Estimate (95% CI)	*p* value	Crude Estimate (95% CI)	*p* value	Adjusted Estimate (95% CI)	*p* value
Total plasma concentration of amodiaquine and desethylamodiaquine, per 750^*^ nmol/litre increase	356	12.98 (8.73 to 17.16)	<0.0001	10.43 (5.92 to 15.00)	<0.0001	20.35 (15.56 to 25.15)	<0.0001	10.67 (6.13 to 15.21)	<0.0001	6.10 (1.80 to 10.38)	0.0055	10.26 (5.71 to 14.81)	<0.0001
Body temperature change, per 1°C increase	356	-5.30 (-7.12 to -3.47)	<0.0001	-3.97 (-6.49 to -1.46)	0.0021	-10.59 (-12.52 to -8.65)	<0.0001	-4.04 (-6.57 to -1.52)	0.0018	-0.374 (-2.25 to 1.50)	0.6948	-3.90 (-6.43 to -1.37)	0.0026
Sex	356												
Female	187	Reference		Reference		Reference		Reference		Reference		Reference	
Male	169	-16.24 (-25.48 to -7.00)	0.0009	-17.35 (-26.62 to -8.08)	0.0004	-12.72 (-22.11 to -3.34)	0.0088	-13.30 (-22.94 to -3.66)	0.0078	-19.54 (-29.00 to -10.08)	0.0001	-21.05 (-30.57 to -11.52)	<0.0001
Age, per 10-year increase	356	2.33 (-0.25 to 0.71)	0.3339	4.16 (-0.17 to 8.49)	0.0595	2.87 (-1.78 to 7.52)	0.2209	4.47 (-0.30 to 8.98)	0.0515	1.78 (-3.32 to 6.88)	0.4859	3.87 (-0.57 to 8.32)	0.0864
RR interval change, per 300-millisecond increase	356	7.50 (4.46 to 10.54)	<0.0001	-0.42 (-4.80 to 3.95)	0.8475	19.69 (16.65 to 22.74)	<0.0001	11.63 (7.25 to 16.01)	<0.0001	-3.69 (-6.75 to -0.65)	0.0177	-11.45 (-15.84 to -7.06)	<0.0001

$QTcS=\frac{QT}{RR^{0.42}}\;\text{\&}\;QTcF=\frac{QT}{\sqrt[{3}]{RR}}\;\text{\&}\;QTcB=\frac{QT}{\sqrt{RR}}$, where RR is in units of seconds

* Mean maximum total plasma drug concentration (rounded) after a 3-day course of amodiaquine from pharmacokinetic analysis of same study

† Mean change in RR interval from baseline (rounded) after last dose of amodiaquine treatment in this study
