# Cardiovascular concentration–effect relationships of amodiaquine and its metabolite desethylamodiaquine: Clinical and preclinical studies

**DOI:** 10.1111/bcp.15569

**Published:** 2022-11-08

**Authors:** Xin Hui S. Chan, Palang Chotsiri, Rebecca A. Capel, James Pike, Borimas Hanboonkunupakarn, Sue J. Lee, Maryam Hanafiah, Yan Naung Win, Maegan A. Cremer, Jean‐René Kiechel, Bernhards Ogutu, Walter R. J. Taylor, Rebecca‐Ann B. Burton, Joel Tarning, Nicholas J. White

**Affiliations:** ^1^ Mahidol‐Oxford Tropical Medicine Research Unit, Faculty of Tropical Medicine Mahidol University Bangkok Thailand; ^2^ Centre for Tropical Medicine and Global Health, Nuffield Department of Medicine University of Oxford Oxford UK; ^3^ Department of Pharmacology University of Oxford Oxford UK; ^4^ Department of Clinical Tropical Medicine, Faculty of Tropical Medicine Mahidol University Bangkok Thailand; ^5^ Department of Preventive and Social Medicine University of Medicine Taunggyi Myanmar; ^6^ Drug for Neglected Diseases Initiative Geneva Switzerland; ^7^ Kenya Medical Research Institute Kisumu Kenya; ^8^ WorldWide Antimalarial Research Network, Centre for Tropical Medicine and Global Health, Nuffield Department of Medicine University of Oxford Oxford UK

**Keywords:** antimalarial, cardiovascular, drug safety, malaria, pharmacodynamics, QT prolongation, quinoline

## Abstract

**Aims:**

Amodiaquine is a 4‐aminoquinoline used extensively for the treatment and prevention of malaria. Orally administered amodiaquine is largely converted to the active metabolite desethylamodiaquine. Amodiaquine can cause bradycardia, hypotension, and electrocardiograph QT interval prolongation, but the relationship of these changes to drug concentrations is not well characterized.

**Methods:**

We conducted a secondary analysis of a pharmacokinetic study of the cardiac safety of amodiaquine (10 mg base/kg/day over 3 days) in 54 Kenyan adults (≥18 years) with uncomplicated malaria. Nonlinear mixed effects modelling was used to assess amodiaquine and desethylamodiaquine concentration–effect relationships for vital sign (pulse rate, blood pressure) and electrocardiograph interval (QT, QRS, PR) outcomes. We also measured the spontaneous beating heart rate after cumulative dosing of amodiaquine and desethylamodiaquine in isolated mouse atrial preparations.

**Results:**

Amodiaquine and desethylamodiaquine caused concentration‐dependent mean decreases in pulse rate (1.9 beats/min per 100 nmol/L; 95% confidence interval: 1.5–2.4), supine systolic blood pressure (1.7 mmHg per 100 nmol/L; 1.2–2.1), erect systolic blood pressure (1.5 mmHg per 100 nmol/L; 1.0–2.0) and erect diastolic blood pressure (1.4 mmHg per 100 nmol/L; 1.0–1.7). The mean QT interval prolongation was 1.4 ms per 100 nmol/L irrespective of correction factor after adjustment for residual heart rate dependency. There was no significant effect of drug concentration on postural change in blood pressure or PR and QRS intervals. In mouse atria, the spontaneous beating rate was significantly reduced by amodiaquine (*n* = 6) and desethylamodiaquine (*n* = 8) at 3 μmol/L (amodiaquine: 10 ± 2%; desethylamodiaquine: 12 ± 3%) and 10 μmol/L (amodiaquine: 50 ± 7%; desethylamodiaquine: 46 ± 6%) concentrations with no significant difference in potency between the 2 compounds.

**Conclusion:**

Amodiaquine and desethylamodiaquine have concentration‐dependent effects on heart rate, blood pressure, and ventricular repolarization.

**What is already known about this subject**

- Amodiaquine is a 4‐aminoquinoline antimalarial used extensively for the treatment and prevention of malaria with an excellent track record of cardiac safety.
- Bradycardia, hypotension, and electrocardiograph QT interval prolongation have been observed after amodiaquine administration in humans and animals.
- A causal role of amodiaquine and its main active metabolite desethylamodiaquine for these cardiovascular effects has been proposed but has yet to be defined.

**What this study adds**

- We characterized the concentration dependency of the bradycardic, hypotensive, and QT prolonging effects of amodiaquine and desethylamodiaquine in clinical and preclinical studies providing evidence of their causal role.
- In a nonlinear mixed effects modelling reanalysis of a cardiac safety study of amodiaquine for uncomplicated malaria, increases in amodiaquine and desethylamodiaquine drug concentrations were associated with mean decreases in pulse rate and blood pressure along with a mean increase in electrocardiograph QT interval prolongation.
- In studies of spontaneously beating murine atrial preparations, amodiaquine and desethylamodiaquine had direct concentration‐dependent bradycardic effects of similar potency.

## 1 INTRODUCTION

Amodiaquine (AQ), a 4‐aminoquinoline structurally similar to chloroquine, is an important antimalarial drug that has been deployed extensively in the treatment and prevention of malaria over the past 70 years. First synthesized by the US World War II antimalarial research programme,^1^, ^2^ it was initially sold as monotherapy under the trade name of Camoquin by the American pharmaceutical company Parke‐Davis.^2^ In 2005, the World Health Organization (WHO) recommended that artemisinin‐based combination therapy should become the first‐line treatment for all falciparum malaria. In 2008, artesunate (AS)–AQ (ASAQ), the fixed‐dose formulation of AQ and AS, became the first major development product of a public‐private partnership, the Drugs for Neglected Diseases initiative. ASAQ is now recommended by the WHO for the treatment of uncomplicated *Plasmodium falciparum* and *Plasmodium vivax* malaria^3^ and is the first‐line oral antimalarial in more than 20 African countries^4^ where malaria is endemic. Since 2012, the WHO has recommended use of seasonal malaria chemoprevention (SMC) for young children (aged 3–59 months) living in areas of seasonal high‐intensity malaria transmission in the Sahel subregion of Africa. SMC comprises a single dose of sulfadoxine–pyrimethamine together with AQ divided over 3 days monthly during the rainy season for up to 4 months annually. Millions of children are now protected with SMC every year.^5^

The cardiovascular effects of AQ have been recognized from the earliest studies in animal models.^1^ During its development, pulsus bigeminus was noted in anaesthetized dogs receiving high doses of parenteral AQ.^1^ Like chloroquine, AQ exhibits anti‐arrhythmic properties, terminating experimental atrial arrhythmias in both decentralized and innervated canine hearts^6^ but, unlike chloroquine, does not appear to protect against experimental ventricular arrhythmias.^7^ Electrocardiograph QT interval prolongation,^7^ bradycardia^8^, and hypotension^8^ have also been observed after parenteral AQ administration to anaesthetized dogs and cats. We have reported recently that AQ prolongs the QT interval less, but is more bradycardic and hypotensive than chloroquine at current standard oral malaria treatment doses when given to adolescents^9^ and adults.^10^ The clinical significance of these cardiovascular effects with standard malaria treatment dosing is unclear^10^ although bradycardia and hypotension may contribute to the higher incidence of mild asthenia and asthenia‐like reactions after AQ compared to other antimalarials.^11^, ^12^, ^13^ Direct multiple ion channel blockade of cardiac^14^, ^15^, ^16^, ^17^, ^18^ and vascular^19^ myocytes along with altered autonomic tone^20^, ^21^ may both be relevant. There are few data on the cardiovascular pharmacology of AQ, and information on its main metabolite desethylamodiaquine is especially limited, as the metabolite was only identified in the 1980s.^22^ This was around the time reports of the fatal toxicity of AQ in chemoprophylaxis^23^, ^24^, ^25^ led to its temporary withdrawal in 1990^26^ from the list of WHO‐recommended antimalarials.

We conducted a secondary analysis of a clinical pharmacokinetic study of ASAQ in adult malaria patients focusing on electrocardiographic interval (RR, QT, QRS and PR) and cardiovascular vital sign (pulse rate and blood pressure) outcomes. This analysis of the clinical study was complemented by laboratory assessment of the concentration–heart rate response in murine atrial preparations with intact sinoatrial node (SAN).

## 2 METHODS

### 2.1 Clinical

#### 2.1.1 Trial design

This was an open‐label randomized controlled trial conducted between 2007 and 2008 in the Chulaimbo Sub‐District Hospital of Kisumu, Kenya. The trial compared the fixed‐dose ASAQ combination with loose (i.e. nonfixed dose) AS + AQ in adult patients (aged 18–60 years inclusive) presenting with acute uncomplicated *P. falciparum* monoinfection with an asexual parasitaemia of >1000 parasites/μL and either a history of fever or a measured temperature of ≥37.5°C in the preceding 24 h. Pregnant or lactating women, those with significant known comorbidities (including severe malnutrition or splenectomy), individuals with an electrocardiograph (ECG) abnormality requiring urgent treatment or those who had taken an artemisinin derivative and/or sulfadoxine–pyrimethamine in the previous 3 or 7 days, respectively, were excluded. Further clinical details are reported in full elsewhere.^27^, ^28^

#### 2.1.2 Drug regimen

Patients received either 2 fixed‐dose ASAQ tablets (100/270 mg, Sanofi‐Aventis, France) daily for a total dose over 3 days of 600 mg of AS and 1620 mg of AQ base or 4 tablets each of nonfixed dose AS (Arsumax 50 mg, Sanofi‐Aventis, France) and AQ (Flavoquine 153 mg, Sanofi‐Aventis, France) daily for a total dose over 3 days of 600 mg of AS and 1836 mg of AQ base. No concomitant food was given. All treatments were directly observed. Patients who vomited a dose within 1 h after drug administration were retreated. Exact dosing times were recorded and used in modelling.

#### 2.1.3 Clinical, parasitological and ECG procedures

Medical and drug histories were taken and physical examinations performed at baseline (D0). Basic clinical assessments including measurement of vital signs (pulse rate, supine and erect blood pressure, and axillary temperature) were conducted, and malaria thick blood film slides were prepared at D0, D1, D2, D3, D7, D14, D21 and D28. A blood film was considered negative if no asexual parasites were seen after examination of 1000 white blood cells. Patients were asked about adverse events of headache, weakness, anorexia, nausea, abdominal pain, itching, vomiting, diarrhoea, rhinitis, cough, vertigo and rash at each clinical assessment. Standard 12‐lead ECGs were performed on D0 (predose, +2 and +4 h), D2 (+2 and +4 h) and D28. ECGs were assessed centrally by cardiologists for arrhythmias and measurement of ECG intervals (RR, QT, QRS and PR).

#### 2.1.4 Blood sampling procedures

Full blood counts were performed on D0 (predose), D7 and D28. Blood samples for drug concentration measurements were taken from all patients at fixed time points on D0 (predose), D7, D14, D21 and D28. Each patient also had additional samples taken at random combinations of the following time points on D0 (+0.25, +0.5, +1, +1.5, +2 and +4 h) and D2 (+0.25, +0.5, +1, +1.5, +2 and +4 h). Exact sample times were recorded and used in the pharmacokinetic modelling.

#### 2.1.5 Pharmacokinetic methods

Venous plasma samples were analysed using liquid chromatography–tandem mass spectrometry methods. AQ, desethylamodiaquine and the internal standard (AST‐D4) were analysed by reversed‐phase liquid chromatography (X Terra C18 MS ‐3.5 μm; 50 × 3 mm id) and tandem mass spectrometry (Sciex API 3000) detection in the Turbo Ion Spray positive mode.

#### 2.1.6 Ethics

The trial protocol was approved by the Kenya Medical Research Institute (KEMRI) Ethical Review Committee. Additional ethical approval for this secondary analysis of fully anonymized individual patient data was not deemed necessary in keeping with University of Oxford Central University Research Ethics Committee guidance.

#### 2.1.7 Data analysis

Trial data were standardized and checked according to a specified data dictionary (Supporting Information) for analysis. Measurements from fixed and nonfixed dose ASAQ arms were pooled as these dose formulations are known to be bioequivalent for AQ and to not have an effect on the pharmacokinetic parameters of these drugs.^28^, ^29^ AS coadministration does not have a significant effect on the bioavailability of AQ.^29^ It is also generally accepted that AS does not have a significant effect on the QT interval.^30^

In view of the inverse relationship between the QT interval and heart rate, measured QT intervals were adjusted for heart rate with the widely used Bazett $\left(\text{QTcB}=\frac{\mathrm{QT}}{\sqrt{\mathrm{RR}}}\right)$ and Fridericia ( $\text{QTcF}=\frac{\mathrm{QT}}{\sqrt[{3}]{\mathrm{RR}}}$) correction formulae. A study‐specific correction formula ( $\text{QTcS}=\frac{\mathrm{QT}}{{\mathrm{RR}}^{0.42}}$) was also applied with the correction exponent derived from log–log linear regression (Supporting Information). The QT interval was analysed as adjusted with the study‐specific (QTcS), Fridericia (QTcF) and Bazett (QTcB) heart rate correction formulae.

All statistical analyses and data visualization were done in R^31^ Version 3.6.0, with linear mixed effects modelling conducted using the *nlme* ^32^ package. Model fit was assessed by visual inspection of residuals, whereas model discrimination was on the basis of likelihood ratio tests with *P* < 0.05 as the threshold for statistical significance.

#### 2.1.8 Pharmacokinetic analysis

Observed AQ and desethylamodiaquine concentrations, transformed into their natural logarithms, were analysed using nonlinear mixed effects modelling implemented in NONMEM Version 7.4^33^ using the first‐order conditional estimation with interactions method. Concentrations below the lower limit of quantification of 1 ng/mL were omitted. In addition to R, Pirana^34^ Version 2.9.0 and Perl‐speaks‐NONMEM (PSN)^35^ Version 4.8.0 were used for automation, model evaluation and diagnostics during the modelling process. The NONMEM $PRIOR functionality was used to stabilize model performance.

The structural pharmacokinetic model of AQ and desethylamodiaquine was based on a model developed from another study of AQ monotherapy for treatment of *P. vivax* malaria in pregnant women with rich (i.e. more intensive) pharmacokinetic sampling^36^ (Figure S1). AQ concentrations were described by lagged first‐order absorption with a 2‐compartment distribution model, followed by a 3‐compartment distribution model of desethylamodiaquine. AQ was assumed to be metabolized completely into desethylamodiaquine as the drug‐metabolite conversion fraction was not identifiable. The final population pharmacokinetic parameter and interindividual variability estimates with their parameter uncertainties from the previous study^36^ were incorporated into the model developed for this study as prior estimates (Supporting Information).

Predicted AQ and desethylamodiaquine concentrations for the time points at which cardiovascular vital signs (pulse rate and blood pressure) and electrocardiographic intervals (RR, QT, QRS and PR) were measured were used in the concentration–effect analyses.

#### 2.1.9 Concentration–effect analyses

Multivariable linear mixed effects modelling was performed with the corrected QT, QRS and PR intervals as well as the change from baseline of pulse rate and blood pressure (systolic and diastolic in the supine and erect positions with their postural differences) as response variables (Supporting Information). Individual patient was the random effect, whereas fixed effect selection was based on directed acyclic graphs of proposed causal relationships among available variables (Figures S2–S5) identified from literature review^37^ and expert consultation.^38^ The drug effect was evaluated using the total predicted concentration of AQ plus its metabolite desethylamodiaquine based on prior evidence that both AQ^6^, ^7^ and desethylamodiaquine^39^ affect cardiovascular physiology as well as lack of evidence for any substantial difference in their activities from descriptive analyses. In the ECG interval models, the other fixed effects were body temperature change, age and sex, with the addition of RR interval change to adjust for residual heart rate changes. For cardiovascular vital signs measured at multiple time points after recovery from malaria, the malaria disease effect was incorporated as a binary categorical fixed effect variable present during Days 0–2 of treatment, with the addition of body temperature change and sex for the change in the pulse rate model only.

### 2.2 Preclinical

#### 2.2.1 Murine atrial studies

Adult male CD‐1 mice (32–37 g, CD‐1 IGS, Charles River Laboratories, UK) were housed maintained in a 12‐h light–dark cycle with ad libitum access to standard diet and sterilized water. Mice were culled by cervical dislocation in accordance with the UK Home Office Guidance on Animals (Scientific Procedures) Act 1986. The heart was excised rapidly and washed in heparin‐containing physiological salt solution (PSS, in millimoles: NaCl 125, NaHCO_3_ 25, KCl 5.4, NaH_2_PO_4_ 1.2, MgCl_2_ 1, glucose 5.5, CaCl_2_ 1.8, oxygenated with 95% O_2_/5% CO_2_). Ventricles were dissected away, the atria were separated at the atrial septum, and the area adjacent to the SAN was cleared of connective tissue. The spontaneously beating right atrial preparation was mounted in a 37°C organ bath containing PSS (continuously oxygenated with 95% O_2_/5% CO_2_) and connected to a force transducer (MLT0201 series, ADInstruments, New Zealand) with a resting tension of 0.2–0.3 g. The tension signal was low‐pass filtered at 20 Hz, and the beating rate calculated from the time interval between contractions (LabChart, ADInstruments, New Zealand). After rate stabilization in PSS (variation in average rate of a 10‐s sample of no more than 2 beats/min over a 10‐min period), cumulative concentrations of AQ dihydrochloride (catalogue number 15314998, Acros Organics, Belgium) or N‐desethylamodiaquine dihydrochloride solution (catalogue number D‐039‐1ML, Sigma Aldrich Co. Ltd., UK) were pipetted directly into the bath. Volume‐ and time‐matched vehicle controls were repetition of the complete experimental protocol using the solvents dimethyl sulfoxide or methanol only. Preparations were excluded if, under control conditions (PSS only), stabilized beating rate was <300 beats/min or arrhythmic. Data are presented as mean ± standard error of the mean. Repeated measures analysis of variance with Tukey's or Dunnett's correction as appropriate was used to assess the isolated heart findings.

## 3 RESULTS

### 3.1 Clinical

Fifty‐four patients aged between 18 and 60 years were randomized. Seven patients had received antimalarial pretreatment: 5 patients with chloroquine and 2 with artemether–lumefantrine. None were receiving any cardiovascular concomitant medications. Baseline characteristics are presented in Table 1.

**TABLE 1:** Baseline characteristics of included population (*n* = 54)

Age (years)	
Median (IQR)	24.0 (19.0–32.0)
18–<35	43 (79.6%)
35–<50	8 (14.8%)
≥50	3 (5.6%)
Weight (kg)	
Median (IQR)	59.0 (53.0–62.0)
Sex	
Female	29 (53.7%)
Male	25 (46.3%)
Temperature (°C)	
Median (IQR)	37.7 (37.1–38.6)
≥37.5	35 (64.8%)
Parasitaemia (parasites/μL)	
Median (IQR)	17 181 (5317–25 876)
>10 000–50 000	24 (44.4%)
>50 000–100 000	6 (11.1%)
>100 000–250 000	1 (1.9%)
Haemoglobin (g/dL)	
Median (IQR)	13.2 (11.9–14.8)
<8	0
Heart rate (beats/min)	
Mean (SD)	91.7 (15.7)^a^
≥140	0
120–<140	2 (3.8%)^a^
100–<120	17 (32.1%)^a^
80–<100	21 (39.6%)^a^
60–<80	13 (24.5%)^a^
<60	0

IQR, interquartile range; SD, standard deviation.

^a^ One participant had missing baseline heart rate.

Fifty‐three completed the full treatment course. One patient in the fixed‐dose ASAQ arm withdrew consent. Four patients were subsequently lost to follow‐up and censored in analyses at the time of dropout. All patients recovered uneventfully. There were no serious cardiovascular events reported in any of the 54 patients.

#### 3.1.1 Pharmacokinetic analysis

A total of 363 postdose venous plasma samples were collected from the 53 participants who received full treatment courses. AQ concentrations were measurable in 115 (31.7%) samples, and 352 (97.0%) had measurable concentrations of desethylamodiaquine (Figure S6).

Population pharmacokinetic parameter estimates were reliable, with small relative standard errors. Secondary pharmacokinetic parameters of maximum concentration, time to maximum concentration, terminal elimination half‐life and total exposure were also computed (Table S1). Goodness‐of‐fit diagnostics and the prediction‐corrected visual predictive checks (Figures S7–S9) demonstrated that the model described the observed data adequately.

#### 3.1.2 Concentration–effect analyses: Cardiovascular vital signs

Plasma concentrations of AQ and desethylamodiaquine were summed. The population mean maximum plasma total concentration (C_max_) of AQ and desethylamodiaquine was approximately 750 nmol/L (or 250 ng/mL; Table S1). This was associated with a mean decrease in pulse rate of 14.6 beats/min (95% confidence interval [CI]: 10.9–18.2). This effect was in addition to independent effects on pulse rate reduction following recovery from fever (5.7 beats/min per 1°C decrease; 95% CI: 3.7–7.6) and from acute malaria (3.0 beats/min; 95% CI: 0.5–5.5). Male sex was not associated with statistically significant effects on pulse rate compared with female sex at this sample size (Table 2).

**TABLE 2:** Multivariable linear mixed effects regression analysis of pulse rate and blood pressure in malaria following treatment with amodiaquine

		*Pulse rate (beats/min)*				*Systolic blood pressure—supine (mmHg)*				*Diastolic blood pressure—erect (mmHg)*			
		Univariable analyses		Multivariable analyses		Univariable analyses		Multivariable analyses		Univariable analyses		Multivariable analyses	
	Number of observations	Crude estimate (95% CI)	*P* value	Adjusted estimate (95% CI)	*P* value	Crude estimate (95% CI)	*P* value	Adjusted estimate (95% CI)	*P* value	Crude estimate (95% CI)	*P* value	Adjusted estimate (95% CI)	*P* value
Total plasma concentration of amodiaquine and desethylamodiaquine, per 750^a^ nmol/L increase	362	−19.51 (−23.10 to −15.92)	<.0001	−14.57 (−18.22 to −10.92)	<.0001	−18.09 (−20.99 to −15.20)	<.0001	−12.43 (−15.95 to −8.92)	<.0001	−10.64 (−13.03 to −8.25)	<.0001	−10.26 (−13.11 to −7.42)	<.0001
Body temperature change, per 1°C increase	362	9.72 (8.32–11.12)	<.0001	5.65 (3.74–7.56)	<.0001	4.46 (3.14–5.79)	<.0001			2.97 (1.94–4.00)	<.0001		
Malaria	362												
Yes (days 0–2)	106	−3.21 (−5.54 to −0.90)	.0066	2.98 (0.50–5.46)	.0186	−9.41 (−11.52 to −7.30)	<.0001	−4.53 (−6.93 to −2.13)	.0002	−1.13 (−2.84 to 0.58)	.1958	2.91 (0.97–4.85)	.0034
No (days 3–28)	256	Reference		Reference		Reference		Reference		Reference		Reference	
Sex	362												
Female	193	Reference		Reference		Reference				Reference			
Male	169	3.06 (−4.81 to 10.93)	.4388	4.12 (−4.17 to 12.41)	.3232	2.52 (−3.74 to 8.78)	.4231			4.14 (−0.80 to 9.08)	.0985		

^a^ Mean maximum total plasma drug concentration (rounded) after a 3‐day course of amodiaquine from pharmacokinetic analysis of same study.

CI, confidence interval.

After adjusting for acute malaria effects, the total plasma concentration of AQ plus desethylamodiaquine at C_max_ was associated with a mean fall of 12.4 mmHg (95% CI: 8.9–15.9) in supine systolic blood pressure and 11.0 mmHg (95% CI: 7.4–14.7) in erect systolic blood pressure. Corresponding reductions in supine diastolic blood pressure were 4.7 mmHg (95% CI: 1.9–7.4), and 10.3 mmHg (95% CI: 7.4–13.1) in erect diastolic blood pressure (Tables 2, S2 and S3). Total drug concentration effects on the postural change between supine and erect blood pressure measurements were small and of unclear significance (Tables S2 and S3).

#### 3.1.3 Concentration–effect analyses: ECG intervals

After adjustment for change in body temperature, age, sex and change in RR interval, the mean‐corrected QT interval prolongation resulting from AQ plus desethylamodiaquine at C_max_ was similar irrespective of the heart rate correction factor used (QTcS: 10.4 ms, 95% CI: 5.9–15.0; QTcF: 10.7 ms, 95% CI: 6.1–15.2; QTcB: 10.3 ms, 95% CI: 5.7–14.8). Unlike the study‐specific corrected QT interval, the Fridericia‐ and Bazett‐corrected QT intervals retained clinically and statistically significant heart rate dependency after adjustment although these were in opposite directions (QTcF: 11.6 ms per 300‐ms increase in RR interval, 95% CI: 7.3–16.0; QTcB: −11.5 ms; 95% CI: −15.8 to −7.1). Thus, without adjustment, use of the Fridericia heart rate correction overestimated AQ‐related QT prolongation from baseline whereas the Bazett correction underestimated it (Table 3).

**TABLE 3:** Multivariable linear mixed effects regression analysis of the corrected QT interval in malaria following treatment with amodiaquine

		*QTcS—Study‐specific correction (ms)*				*QTcF—Fridericia correction (ms)*				*QTcB—Bazett correction (ms)*			
		Univariable analyses		Multivariable analyses		Univariable analyses		Multivariable analyses		Univariable analyses		Multivariable analyses	
	Number of observations	Crude estimate (95% CI)	*P* value	Adjusted estimate (95% CI)	*P* value	Crude estimate (95% CI)	*P* value	Adjusted estimate (95% CI)	*P* value	Crude estimate (95% CI)	*P* value	Adjusted estimate (95% CI)	*P* value
Total plasma concentration of amodiaquine and desethylamodiaquine, per 750^a^ nmol/L increase	356	12.98 (8.73–17.16)	<.0001	10.43 (5.92–15.00)	<.0001	20.35 (15.56–25.15)	<.0001	10.67 (6.13–15.21)	<.0001	6.10 (1.80–10.38)	.0055	10.26 (5.71–14.81)	<.0001
Body temperature change, per 1°C increase	356	−5.30 (−7.12 to −3.47)	<.0001	−3.97 (−6.49 to −1.46)	.0021	−10.59 (−12.52 to −8.65)	<.0001	−4.04 (−6.57 to −1.52)	.0018	−0.374 (−2.25 to 1.50)	.6948	−3.90 (−6.43 to −1.37)	.0026
Sex	356												
Female	187	Reference		Reference		Reference		Reference		Reference		Reference	
Male	169	−16.24 (−25.48 to −7.00)	.0009	−17.35 (−26.62 to −8.08)	.0004	−12.72 (−22.11 to −3.34)	.0088	−13.30 (−22.94 to −3.66)	.0078	−19.54 (−29.00 to −10.08)	.0001	−21.05 (−30.57 to −11.52)	<.0001
Age, per 10‐year increase	356	2.33 (−0.25 to 0.71)	.3339	4.16 (−0.17 to 8.49)	.0595	2.87 (−1.78 to 7.52)	.2209	4.47 (−0.30 to 8.98)	.0515	1.78 (−3.32 to 6.88)	.4859	3.87 (−0.57 to 8.32)	.0864
RR interval change, per 300^b^ ms increase	356	7.50 (4.46–10.54)	<.0001	−0.42 (−4.80 to 3.95)	.8475	19.69 (16.65–22.74)	<.0001	11.63 (7.25–16.01)	<.0001	−3.69 (−6.75 to −0.65)	.0177	−11.45 (−15.84 to −7.06)	<.0001

$\text{QTcS}=\frac{\mathrm{QT}}{{\mathrm{RR}}^{0.42}}\&\text{QTcF}=\frac{\mathrm{QT}}{\sqrt[{3}]{\mathrm{RR}}}\&\text{QTcB}=\frac{\mathrm{QT}}{\sqrt{\mathrm{RR}}},$ where RR is in units of s.

^a^ Mean maximum total plasma drug concentration (rounded) after a 3‐day course of amodiaquine from pharmacokinetic analysis of same study.

^b^ Mean change in RR interval from baseline (rounded) after last dose of amodiaquine treatment in this study.

CI, confidence interval.

There was no significant effect of maximum plasma concentrations of AQ plus desethylamodiaquine on the QRS and PR intervals once adjusted by change in body temperature, sex, age and change in RR interval (QRS: −0.47 ms, 95% CI: −2.51 to 1.57; PR: 2.01 ms; 95% CI: −1.29 to 5.31; Table S4).

### 3.2 Preclinical

#### 3.2.1 Murine atrial studies

**FIGURE 1:**
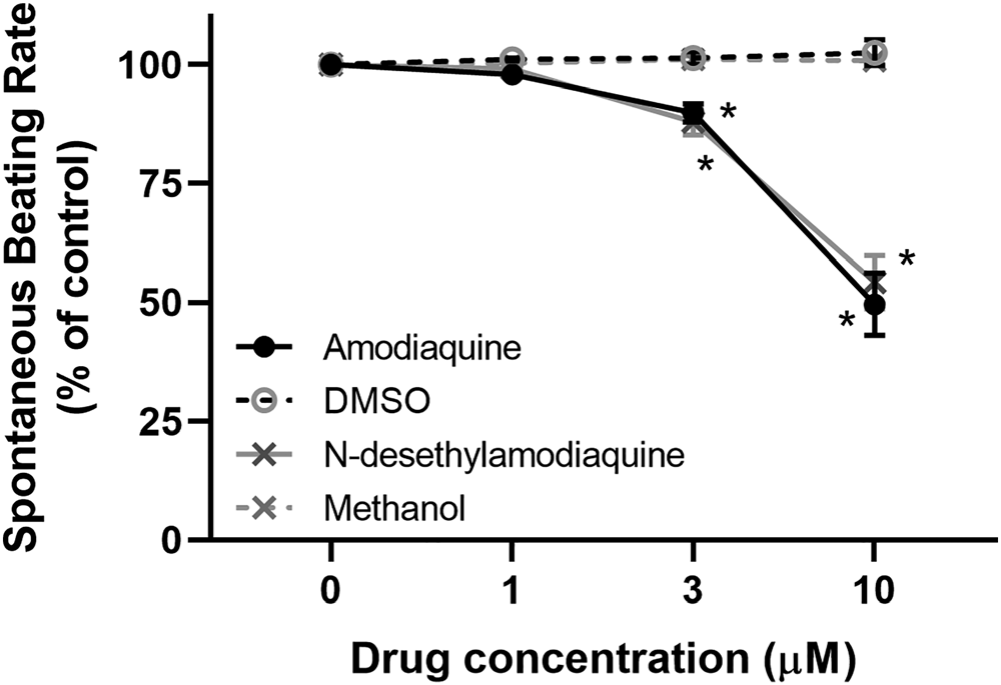
Change (%) in atrial beating rate during cumulative doses of 4‐aminoquinoline antimalarials compared to controls. DMSO, dimethyl sulfoxide; *P < 0.05

Spontaneously beating right atrial preparations contain the intact SAN pacemaker. They can be used to assess the effect of compounds on the intrinsic pacemaker of the heart by measurement of beating rate.^15^ The application of cumulative doses of AQ (*n* = 6) and *N*‐desethylamodiaquine (*n* = 8) to spontaneously beating mouse atrial preparations produced concentration‐dependent reductions in beating rate, which were statistically significant at concentrations of 3 μmol/L (AQ: 10% ± 2%, *P* < 0.01; *N*‐desethylamodiaquine: 12% ± 3%, *P* = .01) and 10 μmol/L (AQ: 50% ± 7%, *P* < .01; *N*‐desethylamodiaquine: 46% ± 6%, *P* < .01) but not 1 μmol/L (AQ: 2% ± 1%, *P* = 0.22; *N*‐desethylamodiaquine: 1% ± 1%, *P* = 0.45) compared with time‐ and volume‐matched controls of the vehicles dimethyl sulfoxide (*n* = 2) and methanol (*n* = 2). There was no statistically significant difference between the response to AQ and N‐desethylamodiaquine at any concentration studied (Figure 1).

## 4 DISCUSSION

The 4‐aminoquinoline antimalarial AQ is a prodrug converted rapidly after oral administration to its active metabolite desethylamodiaquine by cytochrome P450 isozyme 2C8 (CYP2C8).^40^ Thus, desethylamodiaquine, with its higher concentration–time profile and longer terminal elimination half‐life of 9–18 days,^41^ contributes almost all of the antimalarial effect of this widely deployed oral drug in the treatment and prevention of malaria. Although AQ metabolism can vary with CYP2C8 genotype, there is no evidence of the influence of CYP2C8 polymorphisms on the efficacy or safety of AQ.^42^, ^43^

Despite its widespread use, AQ has been relatively little studied in recent years. To our knowledge, this is the most detailed investigation to date of the cardiovascular concentration–effect relationships of AQ and its active metabolite desethylamodiaquine, incorporating both clinical data in malaria patients and a preclinical study of the cardiac pharmacology of desethylamodiaquine.

### 4.1 Cardiovascular vital signs

Bradycardia is a common cardiovascular effect after antimalarial treatment with AQ and mefloquine.^44^ It is observed more in adolescents and adults than in children^10^, ^45^ for reasons which are not fully understood, although modulation of cardiac ion currents by sex hormones may play a role.^46^

Our murine atrial studies show that both AQ and desethylamodiaquine have direct concentration‐dependent bradycardic effects of similar potency. These effects are greater than that of hydroxychloroquine at the concentrations evaluated, measured using the same method in our previous study.^15^ The AQ‐induced bradycardia in both innervated (malaria patients,^9^ anaesthetized dogs^8^) and decentralized (mouse) hearts supports a direct pharmacological effect on cardiac myocyte ion channels through modulation of the pacemaker I_f_ current at the SAN, as observed previously with hydroxychloroquine.^15^ Autonomic tone may also be relevant as both AQ and mefloquine are associated with reversible inhibition of human acetylcholinesterase, with AQ having a much higher potency than mefloquine.^20^, ^21^

The quinoline antimalarials quinine^47^ and chloroquine^48^ are known to cause lethal hypotension when injected rapidly but can be used safely with rate‐controlled continuous intravenous infusion. As proposed for AQ,^19^ these hypotensive effects are likely due to vasodilation and negative inotropy from multiple ion channel blockade.^49^ Orthostatic hypotension is a feature of acute malaria, which is exacerbated by the quinoline antimalarials quinine and mefloquine.^50^ However, in this study of uncomplicated malaria infections, there was no significant effect of the total plasma concentration of AQ and desethylamodiaquine on postural changes in systolic or diastolic blood pressure after adjustment for malaria recovery.

AQ is the most bradycardic of the front‐line antimalarials.^10^ Although AQ and desethylamodiaquine cause concentration‐dependent bradycardia and hypotension in adult malaria patients, the clinical impact of these effects appears to be mild,^27^ although they may contribute to the commonly reported asthenia.

### 4.2 ECG intervals

Drug‐induced QT interval prolongation is the most widely‐used surrogate marker of the risk of development of torsades de pointes (TdP), a polymorphic ventricular tachycardia that can degenerate in some cases into ventricular fibrillation and cause sudden cardiac death.^51^ Despite their QT‐prolonging potential, the front‐line quinoline and structurally related antimalarials recommended currently by the WHO all have excellent track records of cardiac safety. They have not been associated with increased risk of sudden cardiac death or cases of TdP in their extensive use at standard doses for the treatment or prevention of malaria over 7 decades.^10^, ^44^, ^52^

The QT interval lengthens as heart rate decreases. Correction formulae modelling this inverse and nonlinear relationship are used to attempt to minimize the heart rate dependency of measured QT intervals to allow for comparisons across different heart rates. However, the commonly used Bazett and Fridericia corrections are both known to retain significant heart rate dependency,^51^, ^53^ particularly in malaria where there is further confounding from disease recovery that occurs as antimalarial concentrations peak.^10^, ^37^ As before,^10^ a study‐specific correction provided the best heart rate correction of the QT interval (QTcS) in our analysis, although use of the Fridericia (QTcF) and Bazett (QTcB) corrections, respectively, overestimated and underestimated AQ‐related QT prolongation in malaria patients. Once any residual heart rate dependency had been adjusted for the drug‐attributable QT prolongation from AQ and desethylamodiaquine was comparable regardless of correction factor used and consistent with QT prolongation similar to that observed with piperaquine^54^, ^55^ but less than with chloroquine^56^ at standard malaria doses.

In contrast, we found no significant effect of total AQ and desethylamodiaquine concentration on PR or QRS intervals after adjustment for demographic factors (age, sex) and malaria recovery (change in body temperature, change in heart rate). Small increases in unadjusted PR and QRS intervals after AQ for malaria have been previously reported,^57^, ^58^ which may predominantly reflect recovery from malaria rather than a direct drug effect. This differs from chloroquine,^56^ which prolongs both the PR and QRS intervals with >1/4 of the QT prolongation following chloroquine resulting from QRS widening.

### 4.3 Conclusion

The widely used 4‐aminoquinoline AQ, like other quinoline and quinoline‐like drugs, has transient effects on cardiac and vascular physiology. We characterized the concentration dependency of the bradycardic, hypotensive, and QT prolonging effects of AQ and its main metabolite desethylamodiaquine providing further evidence of their causal role. Further characterization of the cardiovascular effects of AQ and desethylamodiaquine in adult healthy volunteers and other preclinical models may help to improve the tolerability of this important medicine in the treatment and prevention of malaria.

## COMPETING INTERESTS

X.H.S.C. is an NIHR Academic Clinical Lecturer in Infectious Diseases at the University of Oxford previously supported by the Medical Research Council of the United Kingdom (MR/N013468/1) and the Jill and Herbert Hunt Travelling Scholarship of the University of Oxford. R.A.C. is a postdoctoral scientist funded by the Wellcome Trust and Royal Society (109371/Z/15/Z). R.A.B.B. is a Senior Research Fellow of Linacre College funded by a Sir Henry Dale Wellcome Trust and Royal Society Fellowship (109371/Z/15/Z) and acknowledges support from the British Heart Foundation (PG/18/4/33521), the Returning Carers' Fund, Medical Sciences Division, University of Oxford, and the Oxford British Heart Foundation Centre for Research Excellence. N.J.W. is a Wellcome Trust Principal Research Fellow (107886/Z/15/Z) and is a recipient of the Bill and Melinda Gates Foundation award (OPP1132628). The Mahidol‐Oxford Tropical Medicine Research Unit research programme is supported by the Wellcome Trust (106698/Z/14/Z). The funders had no role in study design, data collection and analysis, decision to publish or preparation of the manuscript.

## CONTRIBUTORS

X.H.S.C. wrote the first draft of the manuscript. X.H.S.C., P.C., R.A.C., R.A.B.B., J.T. and N.J.W. designed the research. X.H.S.C., P.C., R.A.C., J.P., B.H., S.J.L., M.H., Y.N.W. and M.A.C. performed the analyses and experiments. J.K., B.O. and W.R.J.T. conducted the clinical trial. All authors read and approved the final version of the manuscript.

## Supporting information


**FIGURE S1** Population pharmacokinetic structural model of amodiaquine and desethylamodiaquine^3^
**FIGURE S2** Directed acyclic graph of factors affecting the heart rate in malaria in adults after amodiaquine treatment
**FIGURE S3** Directed acyclic graph of factors affecting blood pressure in malaria after amodiaquine treatment
**FIGURE S4** Directed acyclic graph of factors affecting the electrocardiographic QT interval in malaria after amodiaquine treatment
**FIGURE S5** Directed acyclic graph of factors affecting the electrocardiographic QRS and PR intervals in malaria after amodiaquine treatment
**FIGURE S6** Observed plasma concentrations of amodiaquine and desethylamodiaquine
**FIGURE S7** Goodness‐of‐fit plots for the final pharmacokinetic model
**FIGURE S8** Individual observed and predicted concentrations of amodiaquine and desethyladodi over time
**FIGURE S9** Prediction‐corrected visual predictive check for the final pharmacokinetic model
**TABLE S1** Population pharmacokinetic parameter estimates
**TABLE S3** Factors affecting systolic blood pressure parameters in malaria following treatment with amodiaquine
**TABLE S4** Factors affecting diastolic blood pressure parameters in malaria following treatment with amodiaquine
**TABLE S5** Factors affecting the electrocardiogram QRS and PR intervals in malaria following treatment with amodiaquine

## Data Availability

The data that support the findings of this study are available from the corresponding author upon reasonable request.
