# Supplementary material for: Cardiovascular concentration–effect relationships of amodiaquine and its metabolite desethylamodiaquine: Clinical and preclinical studies
Source: Br J Clin Pharmacol. 2022 Nov 8;89(3):1176–86. doi: 10.1111/bcp.15569 (PMC7614325; doi:10.1111/bcp.15569)
Supplement: Supplementary file 1 — FIGURE S1 Population pharmacokinetic structural model of amodiaquine and desethylamodiaquine3 FIGURE S2 Directed acyclic graph of factors affecting the heart rate in malaria in adults after amodiaquine treatment FIGURE S3 Directed acyclic graph of factors affecting blood pressure in malaria after amodiaquine treatment FIGURE S4 Directed acyclic graph of factors affecting the electrocardiographic QT interval in malaria after amodiaquine treatment FIGURE S5 Directed acyclic graph of factors affecting the electrocardiographic QRS and PR intervals in malaria after amodiaquine treatment FIGURE S6 Observed plasma concentrations of amodiaquine and desethylamodiaquine FIGURE S7 Goodness‐of‐fit plots for the final pharmacokinetic model FIGURE S8 Individual observed and predicted concentrations of amodiaquine and desethyladodi over time FIGURE S9 Prediction‐corrected visual predictive check for the final pharmacokinetic model TABLE S1 Population pharmacokinetic parameter estimates TABLE S3 Factors affecting systolic blood pressure parameters in malaria following treatment with amodiaquine TABLE S4 Factors affecting diastolic blood pressure parameters in malaria following treatment with amodiaquine TABLE S5 Factors affecting the electrocardiogram QRS and PR intervals in malaria following treatment with amodiaquine [file BCP-89-1176-s001.docx]

**Supplementary Appendix**

**Cardiovascular Concentration-Effect Relationships of Amodiaquine and its Metabolite Desethylamodiaquine: Clinical and Pre-clinical Studies**

Xin Hui S Chan, Palang Chotsiri, Rebecca A Capel, James Pike, Borimas Hanboonkunupakarn,
Sue J Lee, Maryam Hanafiah, Yan Naung Win, Maegan A Cremer, Jean-René Kiechel,
Bernhards Ogutu, Walter RJ Taylor, Rebecca-Ann B Burton, Joel Tarning, Nicholas J White

# Supplementary Methods

## Data Standardisation

This was implemented via a bespoke Application Programming Interface in Python version 3.7.2.

### Demographics

#### Age

Age was extracted as standardised to units of years, and otherwise calculated based on the number of years between the subject’s date of birth and the date of the start of the study.

#### Weight

Individual body weight was extracted as standardised to units of kilograms.

### Vital Signs

#### Pulse Rate

Peripheral pulse rate, i.e. heart rate as measured from the peripheral pulse rather than the RR interval on the ECG, was extracted as standardised to units of beats per minute.

#### Blood Pressure

Supine and erect systolic and diastolic blood pressure measurements were standardised to units of mmHg.

#### Body Temperature

Axillary body temperatures were extracted, converted to units of degrees Celsius as required, then standardised by the addition of 0.5°C to original readings^1^.

Body temperature was standardised to units of degrees Celsius using the following formula:

- Temperature (°C) = [Temperature (°F) – 32] / 1.8

### ECG Intervals

#### RR Interval & Heart Rate

RR intervals were standardised to units of milliseconds and transformed into heart rate based on the following formula as necessary:

- Heart rate = 60000/RR interval

#### QT/QTc Interval

Where only corrected QT intervals were available, uncorrected QT intervals were calculated as follows:

- $QT = QTcB*\sqrt{RR}$ as $QTcB =\frac{QT}{\sqrt{RR}}$ (Bazett’s correction formula)
- $QT = QTcF*\sqrt[3]{RR}$ as $QTcF =\frac{QT}{\sqrt[3]{RR}}$ (Fridericia’s correction formula)

where QT intervals are in units of milliseconds and RR intervals are in units of seconds.

#### QRS & PR Intervals

QRS and PR interval measurements were extracted as standardised to units of milliseconds.

### Laboratory Parameters

#### Parasitaemia

The highest malaria parasite density available for each timepoint was extracted.

Malaria parasite count measurements were standardised as parasite density per microlitre of blood according to the following formulae before being logarithmically transformed:

- Parasitaemia = (parasite count per 500 WBC / 500) * WBC count [if WBC count available]
- Parasitaemia = (parasite count per 500 WBC / 500) * 8000 [if WBC count missing]

where WBC counts are in units of mm^3^ of blood

#### Haemoglobin

Haemoglobin was extracted as standardised to units of g/dl.

### Antimalarial Drug-Related Parameters

#### Vomiting & Repeated Doses

Vomiting after dosing and whether the treatment dose was repeated after vomiting were extracted as ‘present’ or ‘absent’. Where a dose was repeated, the date and time of the repeated dose were used to calculate time from dosing.

#### Concomitant Medications

Concomitant medications were extracted as a list of drug names as recorded in the original data if present.

#### Antimalarial Pre-treatment

Antimalarial pre-treatment was extracted as ‘present’ or ‘absent’ with the name of the pre-treatment drug extracted as free text into a separate column where present.

#### Drug Concentrations

Amodiaquine and desethylamodiaquine concentrations in plasma were extracted as standardised to units of nmol/litre.

## Data Integrity Checks

Individual patient data were checked for completeness, as well as for invalid, out-of-range, or inconsistent entries. Values incompatible with what would be observed in malaria clinical trials were considered missing. Queries were raised with study investigators and resolved where possible.

## Data Analysis

### Study-Specific Heart Rate Correction

The correction exponent is the coefficient of $logRR$ from the log-log linear regression:
$logQT \sim logRR + sex + temperature +ECGday*drug+ (1│patient)$

In addition to antimalarial drug, the malaria disease and demographic variables included are those previously identified to have independent effects on the QT interval in malaria in adults^2^.

### Pharmacokinetic Analysis

Figure I: Population Pharmacokinetic Structural Model of Amodiaquine and Desethylamodiaquine^3^

K_a_ = absorption first-order rate constant; V_C(AQ)_ = apparent volume of distribution of the central amodiaquine compartment; V_P(AQ)_ = apparent volume of distribution of the peripheral amodiaquine compartment; Q = inter-compartmental clearance rate of amodiaquine between its central and peripheral compartments; CL­_AQ_ = apparent elimination clearance rate of amodiaquine from its central compartment to form desethylamodiaquine in the metabolite’s central compartment; V_C(DEAQ)_ = apparent volume of distribution of the central desethylamodiaquine compartment; V_P1(DEAQ)_ = apparent volume of distribution of the first peripheral desethylamodiaquine compartment; V_P2(DEAQ)_ = apparent volume of distribution of the second peripheral desthylamodiaquine compartment; Q_1_ = inter-compartmental clearance rate of desethylamodiaquine between its central and first peripheral compartments; Q_2_ = inter-compartmental clearance rate of desethylamodiaquine between its central and second peripheral compartments; CL­_DEAQ_ = apparent elimination clearance rate of desethylamodiaquine from its central compartment

#### Pharmacokinetic Modelling

Pharmacokinetic parameters were assumed to be log-normally distributed. Inter-individual variability was added to all parameters according to the following equation:

$$\theta_{i}=\theta_{p}\times\exp\left( \eta_{i,\theta} \right)$$

where $\theta_{i}$ is the pharmacokinetic parameter estimate for the $i$th individual, $\theta_{p}$ is the population mean value of the investigated parameter, and $\eta_{i,\theta}$ is the deviation of the $i$th individual estimate from the population parameter value. Inter-individual variability was assumed to be normally distributed with mean zero and variance $\omega^{2}$ (diagonal correlation matrix). Where estimates were <10% or had a relative standard error of >50%, inter-individual variability was fixed to zero. The residual unexplained variability in concentration was described by an additive error on the individually predicted logarithmic concentrations which is equivalent to an exponential error for non-transformed concentrations on the arithmetic scale.

Individual body weight, scaled by the median body weight (48.0kg) of the previous study^3^ population, was included as a fixed allometric function to all clearance (power of 0.75) and volume of distribution (power of 1) parameters.

Model discrimination was based on the objective function value (OFV) which is proportional to -2 times the log likelihood of the data and has a Chi-squared distribution. A likelihood ratio test with a reduction in OFV of 3.84 or more was considered significant at *p* = 0.05 for a nested model with a difference of one degree of freedom. Goodness-of-fit plots were used to identify potential model misspecification and systematic errors. Model robustness and parameter confidence intervals were evaluated using a sampling-importance-resampling procedure^4^. Predictive performance was assessed with prediction-corrected visual and numerical predictive checks (n = 2000)^5^.

### Concentration-Effect Analyses

Variable Selection

Variable selection was based on directed acyclic graphs of proposed causal relationships among collected variables informed by literature review and expert consultation used to determine minimal sufficient adjustment sets for regression modelling.

Corrected QT Interval Models

QTcS ~ total drug concentration + Δtemperature + age + sex + ΔRR interval + (1│patient)

QTcF ~ total drug concentration + Δtemperature + age + sex + ΔRR interval + (1│patient)

QTcB ~ total drug concentration + Δtemperature + age + sex + ΔRR interval + (1│patient)

where $QTcS=\frac{QT}{{RR}^{0.42}}$ & $QTcF= \frac{QT}{\sqrt[3]{RR}}$ & $QTcB= \frac{QT}{\sqrt[2]{RR}}$, and RR is in units of seconds

QRS & PR Interval Models

QRS ~ total drug concentration + Δtemperature + age + sex + ΔRR interval + (1│patient)

PR ~ total drug concentration + Δtemperature + age + sex + ΔRR interval + (1│patient)

Change in Pulse Rate Model

ΔHR ~ total drug concentration + malaria + Δtemperature + sex + (1│patient)

Change in Blood Pressure Models

ΔSBP ~ total drug concentration + malaria + (1│patient) [supine]

ΔDBP ~ total drug concentration + malaria + (1│patient) [supine]

ΔSBPe ~ total drug concentration + malaria + (1│patient) [erect]

ΔDBPe ~ total drug concentration + malaria + (1│patient) [erect]

ΔpSBP ~ total drug concentration + malaria + (1│patient) [postural drop = supine - erect]

ΔpDBP ~ total drug concentration + malaria + (1│patient) [postural drop = supine - erect]

Figure II: Directed Acyclic Graph of Factors Affecting the Heart Rate in Malaria in Adults after Amodiaquine Treatment


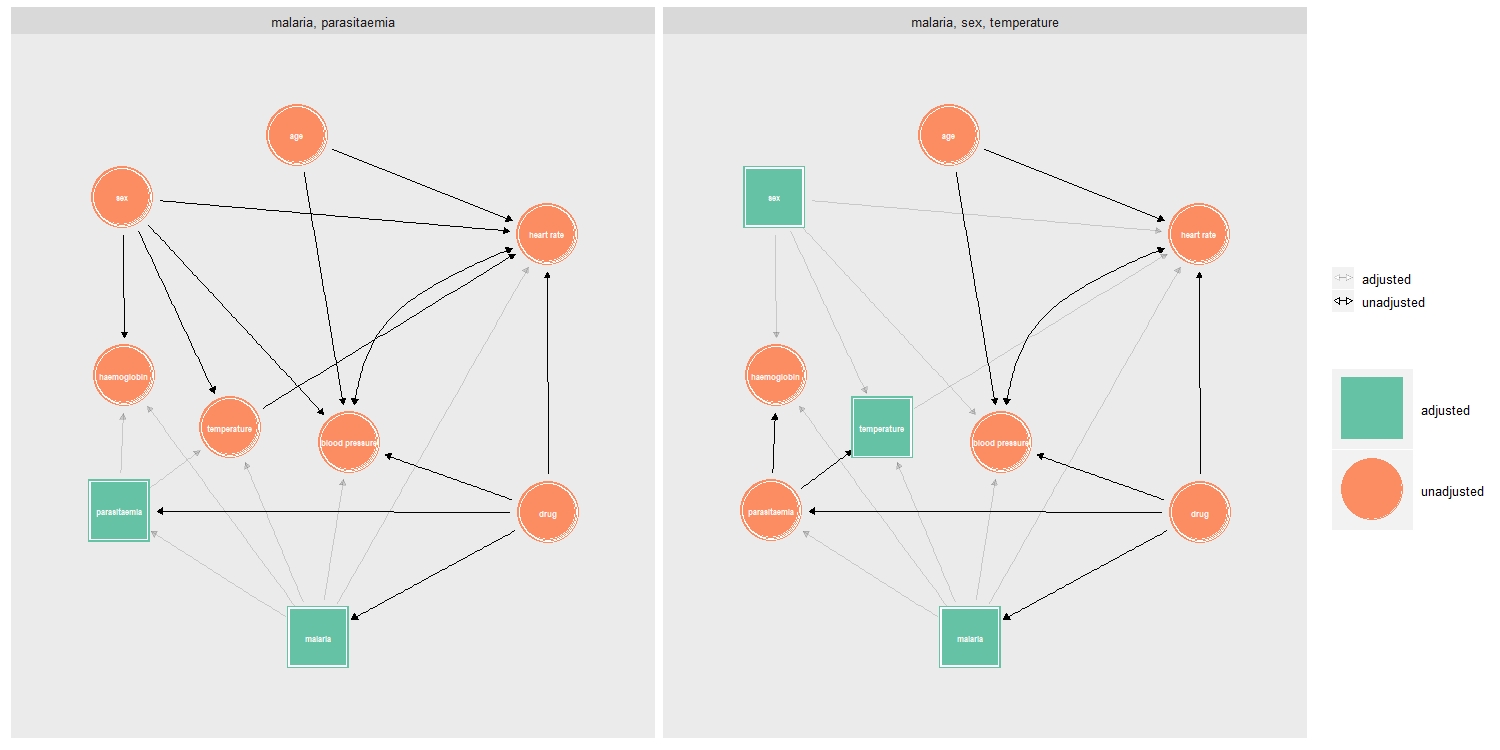


Directed acyclic graph generated in DAGitty^6^ describing proposed causal relationships among factors affecting the heart rate in malaria in adults after antimalarial treatment with amodiaquine showing minimal sufficient covariate adjustment set (facet label & green squares). Bidirectional arrows do not represent reciprocal causation but depict unobserved confounders. The minimal adjustment set consisting of disease variables of malaria and temperature along with the demographic covariate of sex were included as fixed effects in multivariable linear mixed effects analyses.

Figure III: Directed Acyclic Graph of Factors Affecting Blood Pressure in Malaria After Amodiaquine Treatment


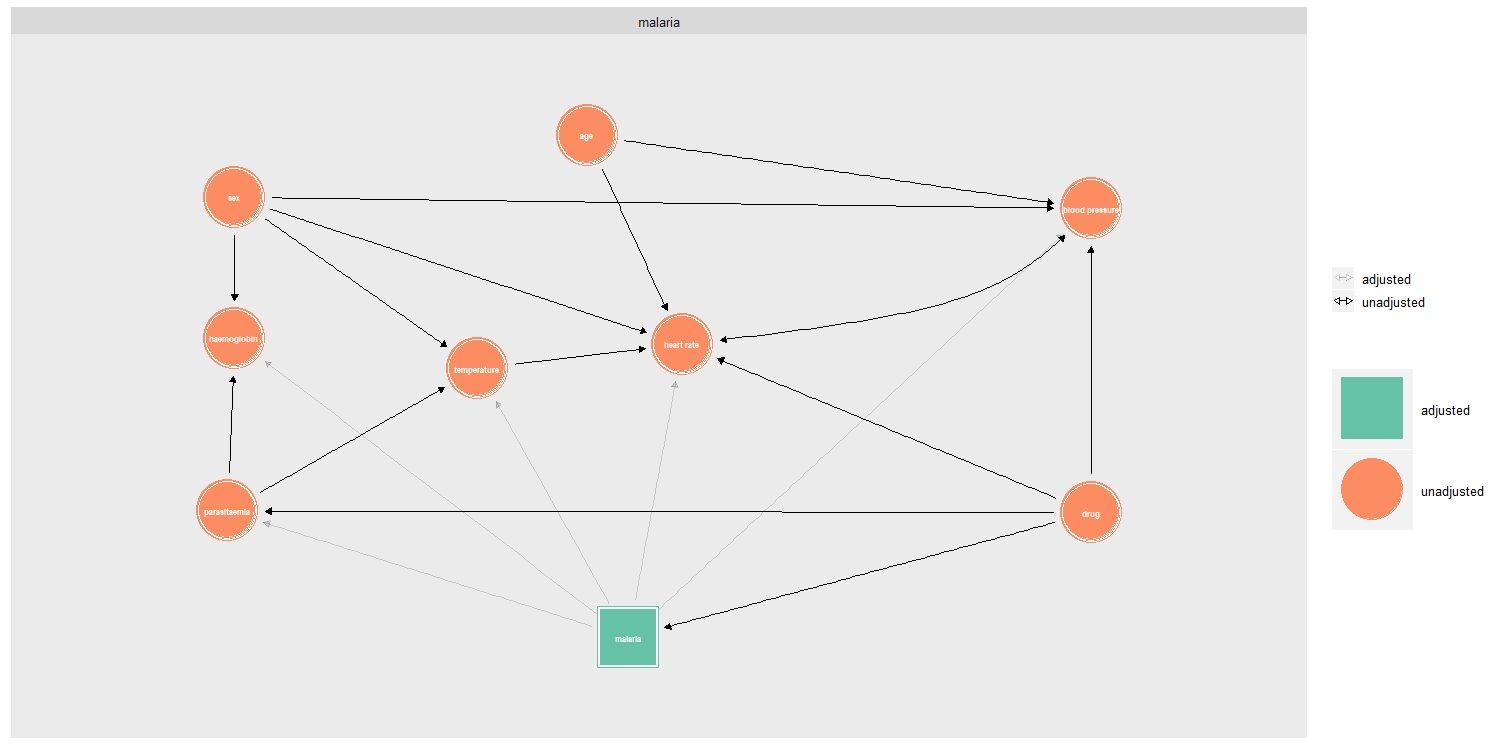


Directed acyclic graph generated in DAGitty^6^ describing proposed causal relationships among factors affecting blood pressure in malaria after antimalarial treatment with amodiaquine showing minimal sufficient covariate adjustment set (facet label & green squares). The minimal adjustment set consisting of the malaria disease variable was included as a fixed effect in multivariable linear mixed effects analyses.

Figure IV: Directed Acyclic Graph of Factors Affecting the Electrocardiographic QT Interval in Malaria after Amodiaquine Treatment

**
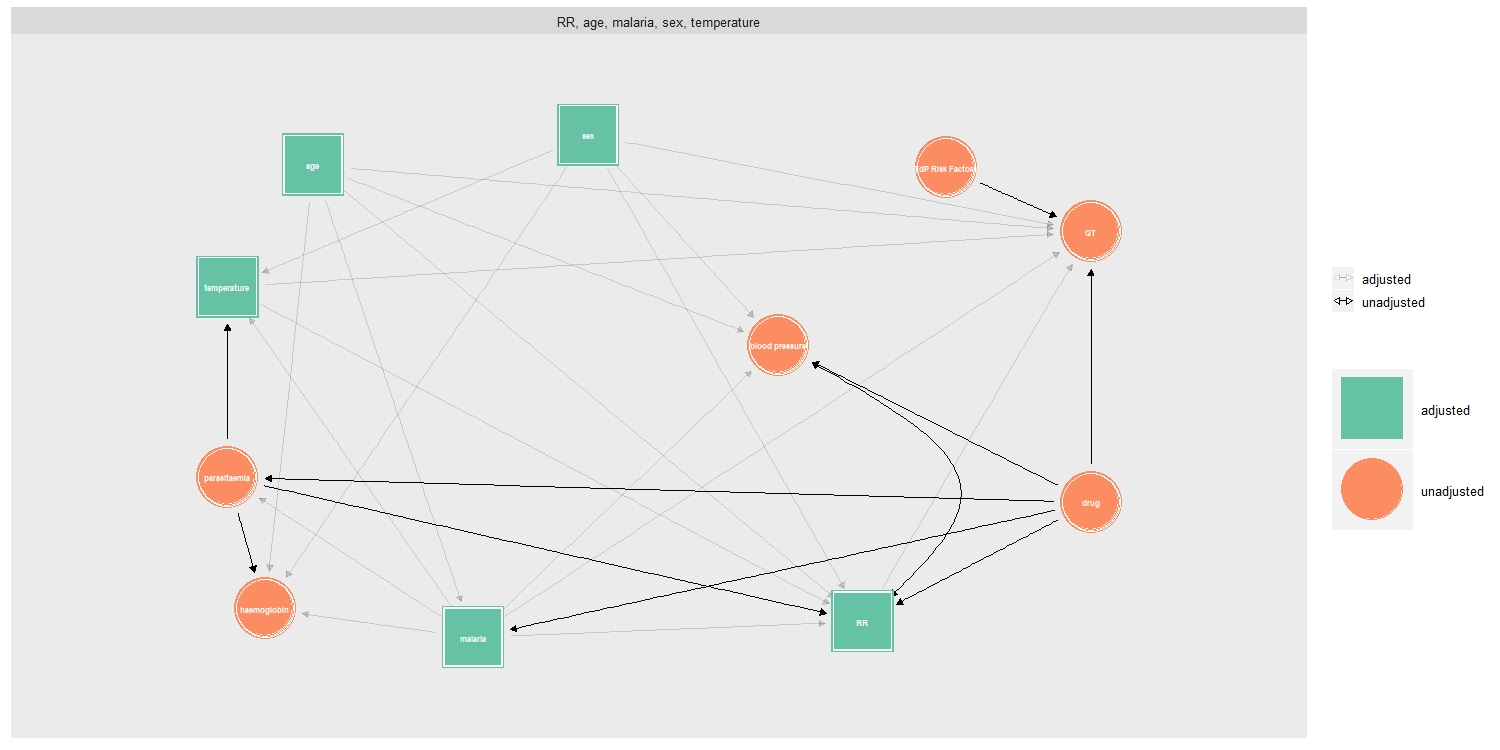
**

Directed acyclic graph generated in DAGitty^6^ describing proposed causal relationships among factors affecting the electrocardiographic QT interval in malaria after antimalarial treatment with amodiaquine showing minimal sufficient covariate adjustment set (facet label & green squares). Bidirectional arrows do not represent reciprocal causation but depict unobserved confounders. The minimal adjustment set consisting of malaria disease variables of malaria and temperature along with demographic covariates of age and sex were included as fixed effects in multivariable linear mixed effects analyses. A study-specific heart rate correction factor was used for RR interval-related confounding.

Figure V: Directed Acyclic Graph of Factors Affecting the Electrocardiographic QRS & PR Intervals in Malaria after Amodiaquine Treatment


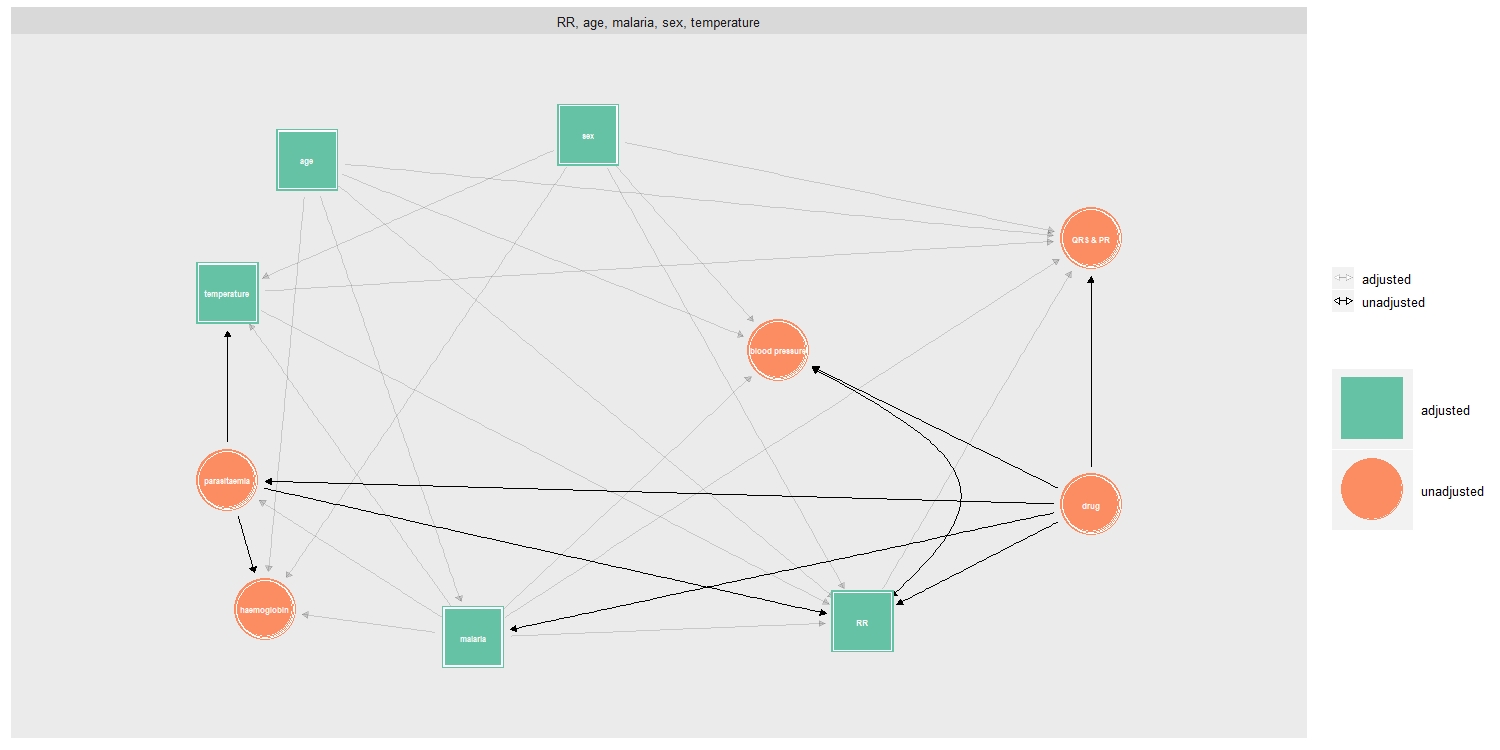


Directed acyclic graph generated in DAGitty^6^ describing proposed causal relationships among factors affecting the electrocardiographic QRS and PR intervals in malaria after antimalarial treatment with amodiaquine showing minimal sufficient covariate adjustment set (facet label & green squares). Bidirectional arrows do not represent reciprocal causation but depict unobserved confounders. The minimal adjustment set consisting of malaria disease variables of malaria and temperature along with demographic covariates of age and sex were included as fixed effects in multivariable linear mixed effects analyses. A study-specific heart rate correction factor was used for RR interval-related confounding.

# Supplementary Results

## Pharmacokinetic Analysis

Figure VI: Observed Plasma Concentrations of Amodiaquine and Desethylamodiaquine


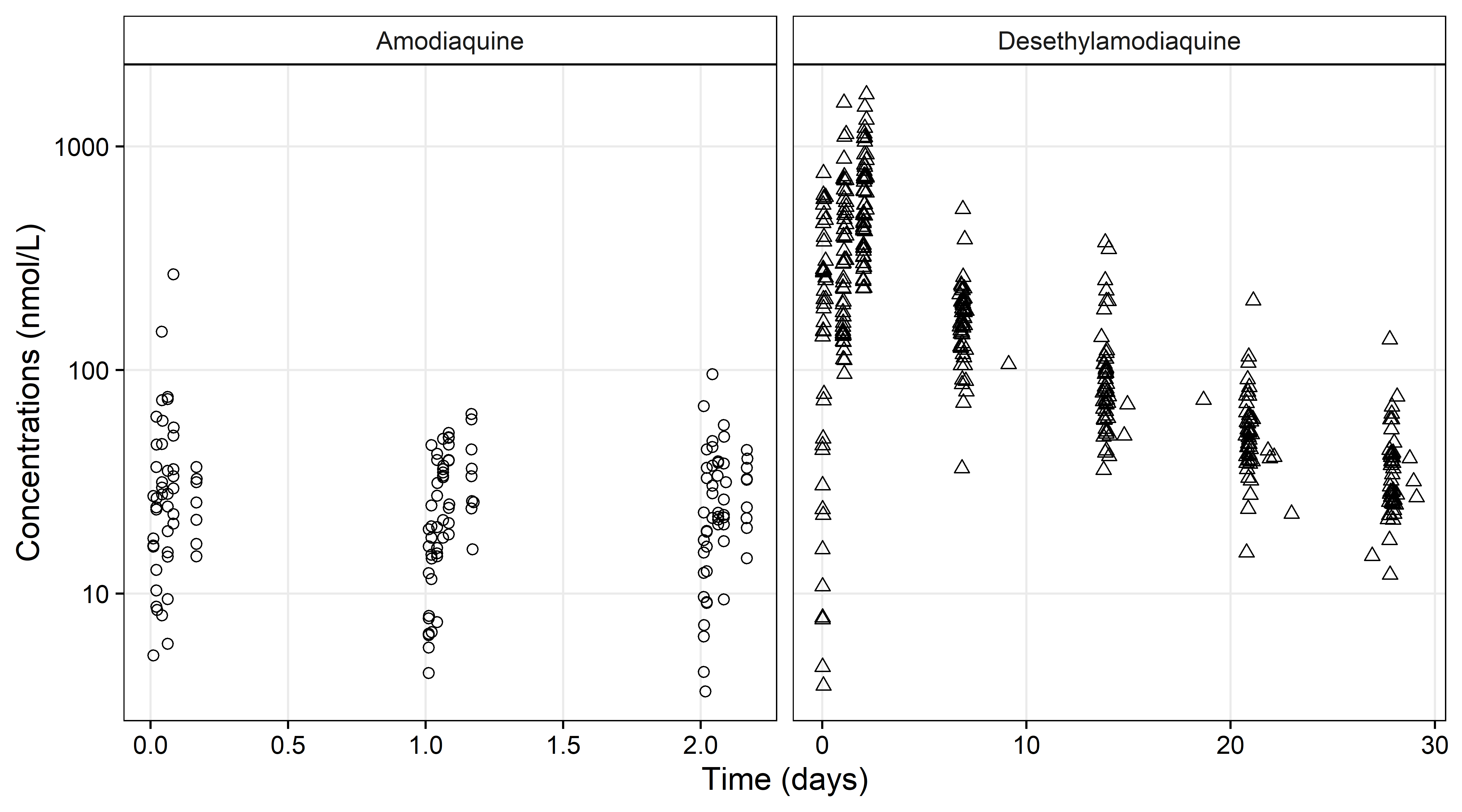


Observed plasma concentrations of amodiaquine (open circles) and desethylamodiaquine (open triangles) from sparse sampling

Table I: Population Pharmacokinetic Parameter Estimates

| **Population Pharmacokinetic Parameters** | **Prior Estimates^*^** | **Population Estimates^†^** | **95% Confidence Interval^‡^** | **%RSE^‡^** |
| --- | --- | --- | --- | --- |
| *Amodiaquine* | | | | |
| K_a_ (hour^-1^) | 0.418 | 0.469 | 0.410–0.508 | 5.45% |
| Absorption lag time (hour) | 0.314 | 0.226 | 0.214–0.233 | 2.28% |
| V_C(AQ)_ (litres) | 3520 | 5840 | 4520–7110 | 11.1% |
| CL_(AQ)_ (litres/hour) | 2420 | 2790 | 2510–3070 | 4.80% |
| V_P(AQ)_ (litres) | 32500 | 34200 | 29600–40100 | 7.85% |
| Q_(AQ)_ (litres/hour) | 2470 | 2900 | 2400–3350 | 8.09% |
| σ_AQ_ | NA | 0.108 | 0.0763–0.145 | 8.42% |
| *Desethylamodiaquine* | | | | |
| V_C(DEAQ)_ (litres) | 198 | 213 | 158–272 | 13.6% |
| CL_(DEAQ)_ (litres/hour) | 34.1 | 35.2 | 33.3–37.8 | 3.25% |
| V_P1(DEAQ)_ (litres) | 2630 | 2760 | 2310–3180 | 7.79% |
| Q_P1(DEAQ)_ (litres/hour) | 163 | 181 | 157–206 | 13.6% |
| V_P2(DEAQ)_ (litres) | 5650 | 6530 | 5740–7410 | 6.24% |
| Q_P2(DEAQ)_ (litres/hour) | 25.4 | 30 | 25.6–35.2 | 7.98% |
| σ_DEAQ_ | NA | 0.255 | 0.216–0.304 | 4.49% |
| *Inter-Individual Variability (%CV)* | | | | |
| Absorption lag time (hour) | 0.359 (65.7%) | 0.541 (73.6%) | 0.342–0.738 | 9.03% |
| V_C(AQ)_ (litres) | 0.382 (68.2%) | 0.772 (87.9%) | 0.383–1.34 | 16.1% |
| CL_(AQ)_ (litres/hour) | 0.0578 (24.4%) | 0.0586 (24.2%) | 0.0368–0.0890 | 12.5% |
| V_P(AQ)_ (litres) | 0.0603 (24.9%) | 0.0612 (27.4%) | 0.0337–0.0814 | 10.0% |
| Q_(AQ)_ (litres/hour) | 0.0678 (26.5%) | 2.94 (171%) | 1.95–4.56 | 11.4% |
| V_C(DEAQ)_ (litres) | 0.196 (46.5%) | 0.691 (83.1%) | 0.335–1.08 | 13.4% |
| CL_(DEAQ)_ (litres/hour) | 0.0522 (23.1%) | 0.0373 (19.3%) | 0.0255–0.0532 | 9.73% |
| V_P2(DEAQ)_ (litres) | 0.0239 (15.6%) | 0.0211 (14.5%) | 0.0132–0.0298 | 9.87% |
| F | 0.0251 (15.9%) | 0.0256 (16.0%) | 0.0164–0.0385 | 10.8% |
| *Secondary Parameters* | | | | |
| C_max(AQ)_ (ng/ml) |  | 13.8 | 7.86-30.6 |  |
| C_max(AQ)_ (nmol/litre) |  | 42.1 | 24.0-93.3 |  |
| T_max(AQ)_ (hours) |  | 1.70 | 0.793-3.56 |  |
| t_1/2(AQ)_ (hours) |  | 5.93 | 0.861-72.1 |  |
| AUC_7 days_ (hours x ng/ml) |  | 494 | 341-681 |  |
| C_max(DEAQ)_ (ng/ml) |  | 240 | 166-429 |  |
| C_max(DEAQ)_ (nmol/litre) |  | 731 | 506-1310 |  |
| T_max(DEAQ)_ (hours) |  | 3.61 | 2.00-6.67 |  |
| t_1/2(DEAQ)_ (days) |  | 13.4 | 11.8-15.0 |  |
| AUC_28 days_ (hours x μg/ml) |  | 31.7 | 19.8-46.3 |  |

AQ = amodiaquine; DEAQ = desethylamodiaquine; K_a_ = absorption first-order rate constant; V_C_ = apparent volume of distribution of central compartment; CL­ = apparent elimination clearance rate from central compartment; V_P_ = apparent volume of distribution of peripheral compartment; Q = inter-compartmental clearance rate between central and peripheral compartment(s); σ = residual error variance; F = relative bioavailability; %CV = coefficient of variation for inter-individual variability computed as $100\times\sqrt{\exp\left( \omega^{2} \right)-1}$; C_max_ = maximum concentration; T_max_ = time to maximum concentration; t_1/2_ = terminal elimination half-life; AUC = area under curve, i.e. total drug exposure; ^*^Population pharmacokinetic estimates with corresponding parameter uncertainties from model developed from a separate study with rich pharmacokinetic sampling^3^; ^†^_­_Population mean estimates from NONMEM® for a ‘typical’ 48.0kg adult patient with uncomplicated malaria; ^‡^From sampling-importance-resampling procedure^4^ of the final model.

Figure VII: Goodness-of-Fit Plots for the Final Pharmacokinetic Model


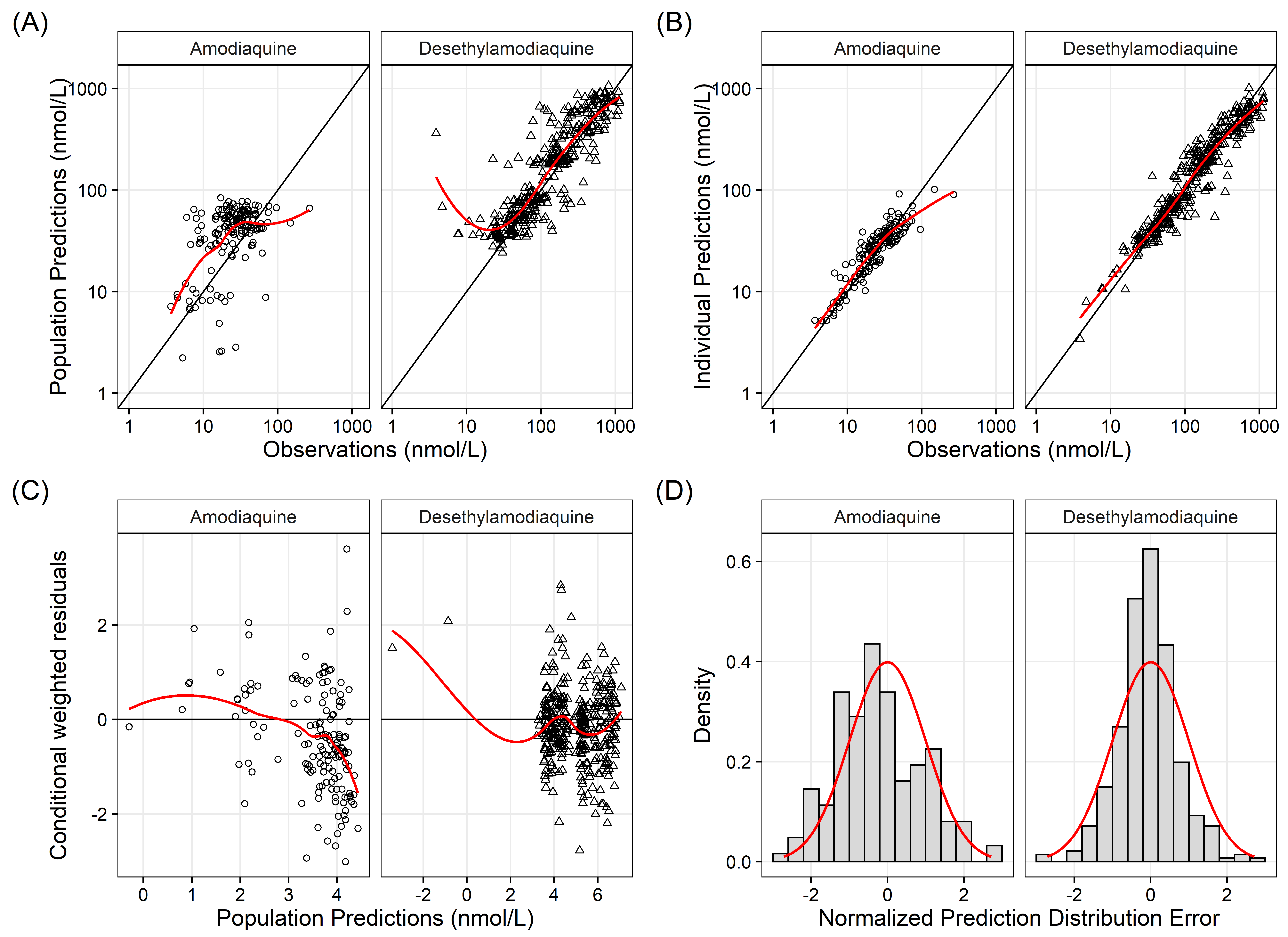


(A) Population predicted plasma concentrations versus observations, (B) Individual predicted concentrations versus observations, (C) Conditional weighted residuals versus population predicted concentrations, (D) Normalised prediction distribution error. In (A), (B), and (C), the solid black line is the identity line, and the solid red line is the locally weighted least squares regression line.

Figure VIII: Individual Observed and Predicted Concentrations of Amodiaquine and Desethyladodi over Time


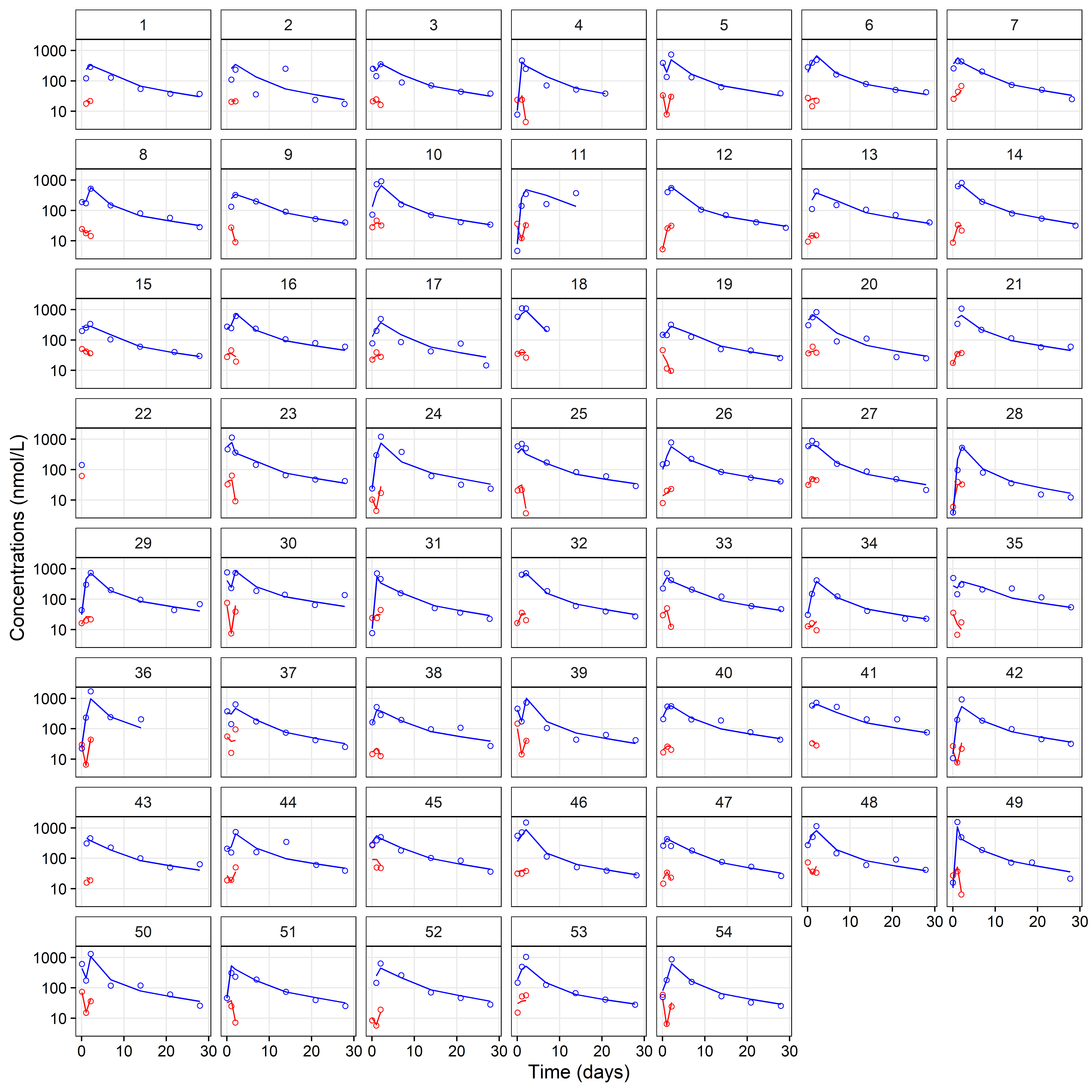


Observed (open circles) and predicted (solid lines) plasma concentrations for amodiaquine (red) and desthyl-amodiaquine (blue) over time for each individual patient (facet label)

Figure IX: Prediction-Corrected Visual Predictive Check for the Final Pharmacokinetic Model


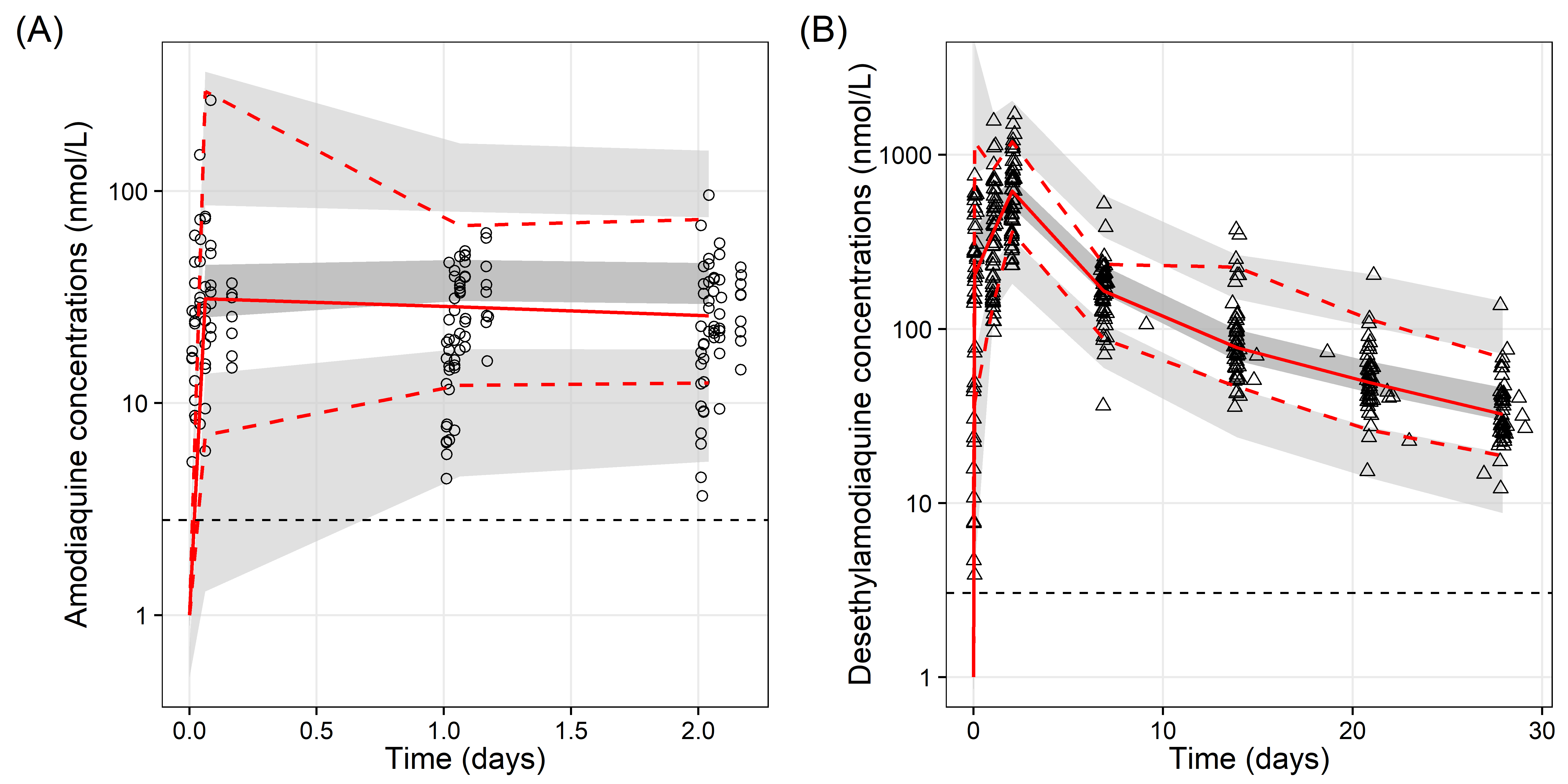


Visual predictive checks for the final pharmacokinetic model of (A) amodiaquine (open circles) and (B) desethylamodiaquine (open triangles) based on 2,000 stochastic simulations. Solid lines represent the median/50^th^ percentile, while dashed lines represent the 5^th^ and 95^th^ percentiles of observed plasma concentrations. Shaded areas represent the 95% confidence intervals around the simulated 5^th^, 50^th^, and 95^th^ percentiles.

**Concentration-Effect Analyses**

Table III: Factors Affecting Systolic Blood Pressure Parameters in Malaria Following Treatment with Amodiaquine

|  |  | ***Systolic Blood Pressure – Erect (mmHg)*** | | | | ***Systolic Blood Pressure – Postural Change (mmHg)*** | | | |
| --- | --- | --- | --- | --- | --- | --- | --- | --- | --- |
|  |  | **Univariable Analyses** | | **Multivariable Analyses** | | **Univariable Analyses** | | **Multivariable Analyses** | |
|  | **Number of Observations** | **Crude Estimate (95% CI)** | ***p* value** | **Adjusted Estimate (95% CI)** | ***p* value** | **Crude Estimate (95% CI)** | ***p* value** | **Adjusted Estimate (95% CI)** | ***p* value** |
| Total plasma concentration of amodiaquine and desethylamodiaquine,  per 750^*^ nmol/l increase | 362 | -12.33 (-15.21 to -9.46) | <0.0001 | -11.05 (-14.75 to -7.35) | <0.0001 | -5.01 (-8.06 to -1.97) | 0.0013 | -0.38 (-4.32 to 3.55) | 0.849 |
| Body temperature change,  per 1°C increase | 362 | 3.00  (1.76 to 4.24) | <0.0001 |  |  | 0.52 (-0.43 to 1.47) | 0.2787 |  |  |
| Malaria | 362 |  |  |  |  |  |  |  |  |
| Yes (days 0, 1, 2) | 106 | -4.73 (-6.91 to -2.55) | <0.0001 | -0.39 (-2.92 to 2.14) | 0.7617 | -4.50 (-6.77 to -2.23) | 0.0001 | -4.35 (-7.10 to -1.59) | 0.0021 |
| No (days 3, 7, 14, 21, 28) | 256 | Reference |  | Reference |  | Reference |  | Reference |  |

^*^Mean maximum total plasma drug concentration (rounded) after a 3-day course of amodiaquine from pharmacokinetic analysis of same study

Table IV: Factors Affecting Diastolic Blood Pressure Parameters in Malaria Following Treatment with Amodiaquine

|  |  | ***Diastolic Blood Pressure – Supine (mmHg)*** | | | | ***Diastolic Blood Pressure – Postural Change (mmHg)*** | | | |
| --- | --- | --- | --- | --- | --- | --- | --- | --- | --- |
|  |  | **Univariable Analyses** | | **Multivariable Analyses** | | **Univariable Analyses** | | **Multivariable Analyses** | |
|  | **Number of Observations** | **Crude Estimate (95% CI)** | **p value** | **Adjusted Estimate (95% CI)** | **p value** | **Crude Estimate (95% CI)** | **p value** | **Adjusted Estimate (95% CI)** | **p value** |
| Total plasma concentration of amodiaquine and desethylamodiaquine,  per 750^*^ nmol/l increase | 362 | -7.97  (-10.25 to -5.69) | <0.0001 | -4.65 (-7.37 to -1.94) | 0.0008 | 2.31 (-0.10 to 4.71) | 0.0602 | 4.87 (1.90 to 7.84) | 0.0014 |
| Body temperature change,  per 1°C increase | 362 | 2.08  (1.11 to 3.05) | <0.0001 |  |  | -0.50  (-1.14 to 0.15) | 0.1331 |  |  |
| Malaria | 362 |  |  |  |  |  |  |  |  |
| Yes (days 0, 1, 2) | 106 | -2.17  (-3.71 to -0.63) | 0.0058 | -0.35  (-2.20 to 1.51) | 0.7141 | -1.00  (-2.75 to 0.75) | 0.2621 | -2.94  (-5.03 to -0.85) | 0.0059 |
| No (days 3, 7, 14, 21, 28) | 256 | Reference |  | Reference |  | Reference |  | Reference |  |

^*^Mean maximum total plasma drug concentration (rounded) after a 3-day course of amodiaquine from pharmacokinetic analysis of same study

Table V: Factors Affecting the Electrocardiogram QRS and PR Intervals in Malaria Following Treatment with Amodiaquine

|  |  | ***QRS Interval (milliseconds)*** | | | | ***PR Interval (milliseconds)*** | | | |
| --- | --- | --- | --- | --- | --- | --- | --- | --- | --- |
|  |  | **Univariable Analyses** | | **Multivariable Analyses** | | **Univariable Analyses** | | **Multivariable Analyses** | |
|  | **Number of Observations** | **Crude Estimate (95% CI)** | **p value** | **Adjusted Estimate (95% CI)** | **p value** | **Crude Estimate (95% CI)** | **p value** | **Adjusted Estimate (95% CI)** | **p value** |
| Total plasma concentration of amodiaquine and desethylamodiaquine,  per 750^*^ nmol/l increase | 356 | 2.34  (0.38 to 4.29) | 0.0194 | -0.47  (-2.51 to 1.57) | 0.6525 | 6.68  (3.51 to 9.84) | <0.0001 | 2.01  (-1.29 to 5.31) | 0.2313 |
| Body temperature change,  per 1°C increase | 356 | -2.08  (-2.86 to -1.30) | <0.0001 | -0.30  (-1.42 to 0.82) | 0.5953 | -3.86  (-5.15 to -2.57) | <0.0001 | -0.54 (-2.38 to 1.30) | 0.5647 |
| Sex | 356 |  |  |  |  |  |  |  |  |
| Female | 187 | Reference |  | Reference |  | Reference |  | Reference |  |
| Male | 169 | 5.44 (0.68 to 10.2) | 0.0259 | 5.09 (0.27 to 9.91) | 0.0388 | 1.60  (-11.84 to 15.04) | 0.8124 | -0.06  (-13.72 to 13.62) | 0.9935 |
| Age,  per 10-year increase | 356 | 1.53 (-0.76 to 3.82) | 0.186 | 1.08 (-1.17 to 3.34) | 0.3386 | 4.03  (-2.17 to 10.22) | 0.1976 | 4.11  (-2.29 to 10.51) | 0.2027 |
| RR interval change,  per 300 millisecond increase | 356 | 4.43 (3.15 to 5.71) | <0.0001 | 4.20  (2.22 to 6.18) | <0.0001 | 8.18  (6.10 to 10.26) | <0.0001 | 6.91  (3.68 to 10.14) | <0.0001 |

^*^Mean maximum total plasma drug concentration (rounded) after a 3-day course of amodiaquine from pharmacokinetic analysis of same study
^†^Mean change in RR interval from baseline (rounded) after last dose of amodiaquine treatment in this study

# References

1. Chue AL, Moore RL, Cavey A, et al. Comparability of tympanic and oral mercury thermometers at high ambient temperatures. *BMC Res Notes* 2012; **5**: 356.

2. Chan XHS, Win YN, Haeusler IL, et al. Factors affecting the electrocardiographic QT interval in malaria: A systematic review and meta-analysis of individual patient data. *PLoS Med* 2020; **17**(3): e1003040.

3. Tarning J, Chotsiri P, Jullien V, et al. Population pharmacokinetic and pharmacodynamic modeling of amodiaquine and desethylamodiaquine in women with Plasmodium vivax malaria during and after pregnancy. *Antimicrob Agents Chemother* 2012; **56**(11): 5764-73.

4. Dosne AG, Bergstrand M, Karlsson MO. An automated sampling importance resampling procedure for estimating parameter uncertainty. *J Pharmacokinet Pharmacodyn* 2017; **44**(6): 509-20.

5. Bergstrand M, Hooker AC, Wallin JE, Karlsson MO. Prediction-corrected visual predictive checks for diagnosing nonlinear mixed-effects models. *AAPS J* 2011; **13**(2): 143-51.

6. Textor J, van der Zander B, Gilthorpe MS, Liskiewicz M, Ellison GT. Robust causal inference using directed acyclic graphs: the R package 'dagitty'. *Int J Epidemiol* 2016; **45**(6): 1887-94.
