# Supplementary material for: Cardiovascular Concentration-Effect Relationships of Amodiaquine and its Metabolite Desethylamodiaquine: Clinical and Pre-clinical Studies
Source: Br J Clin Pharmacol. Author manuscript; Available in PMC 2023 Mar 16. (PMC7614325; doi:10.1111/bcp.15569)
Supplement: Supplementary file 1 [file EMS171002-supplement-Supplementary_Appendix.pdf]

SUPPLEMENTARY APPENDIX

Cardiovascular Concentration-Effect Relationships of Amodiaquine and its Metabolite Desethylamodiaquine: Clinical and Pre-clinical Studies

Xin Hui S Chan, Palang Chotsiri, Rebecca A Capel, James Pike, Borimas Hanboonkunupakarn,

Sue J Lee, Maryam Hanafiah, Yan Naung Win, Maegan Cremer, Jean-René Kiechel,

Bernhards Ogutu, Walter RJ Taylor, Rebecca-Ann B Burton, Joel Tarning, Nicholas J White

1 Supplementary Methods

1.1 DATA STANDARDISATION

This was implemented via a bespoke Application Programming Interface in Python version 3.7.2.

1.1.1 Demographics

1.1.1.1 Age

Age was extracted as standardised to units of years, and otherwise calculated based on the number of years between the subject's date of birth and the date of the start of the study.

1.1.1.2 Weight

Individual body weight was extracted as standardised to units of kilograms.

1.1.2 Vital Signs

1.1.2.1 Pulse Rate

Peripheral pulse rate, i.e. heart rate as measured from the peripheral pulse rather than the RR interval on the ECG, was extracted as standardised to units of beats per minute.

1.1.2.2 Blood Pressure

Supine and erect systolic and diastolic blood pressure measurements were standardised to units of mmHg.

1.1.2.3 Body Temperature

Axillary body temperatures were extracted, converted to units of degrees Celsius as required, then standardised by the addition of 0.5°C to original readings[56].

Body temperature was standardised to units of degrees Celsius using the following formula:

- Temperature (°C) = [Temperature (°F) – 32] / 1.8

1.1.3 ECG Intervals

1.1.3.1 RR Interval & Heart Rate

RR intervals were standardised to units of milliseconds and transformed into heart rate based on the following formula as necessary:

- Heart rate = 60000/RR interval

1.1.3.2 QT/QTc Interval

Where only corrected QT intervals were available, uncorrected QT intervals were calculated as follows:

- $QT = QTcB * \sqrt{RR}$ as $QTcB = \frac{QT}{\sqrt{RR}}$ (Bazett's correction formula)
- $QT = QTcF * \sqrt[3]{RR}$ as $QTcF = \frac{QT}{\sqrt[3]{RR}}$ (Fridericia's correction formula)

where QT intervals are in units of milliseconds and RR intervals are in units of seconds.

1.1.3.3 QRS & PR Intervals

QRS and PR interval measurements were extracted as standardised to units of milliseconds.

1.1.4 Laboratory Parameters

1.1.4.1 Parasitaemia

The highest malaria parasite density available for each timepoint was extracted.

Malaria parasite count measurements were standardised as parasite density per microlitre of blood according to the following formulae before being logarithmically transformed:

- Parasitaemia = (parasite count per 500 WBC / 500) * WBC count [if WBC count available]
- Parasitaemia = (parasite count per 500 WBC / 500) * 8000 [if WBC count missing]

where WBC counts are in units of mm^3 of blood

1.1.4.2 Haemoglobin

Haemoglobin was extracted as standardised to units of g/dl.

1.1.5 Antimalarial Drug-Related Parameters

1.1.5.1 Vomiting & Repeated Doses

Vomiting after dosing and whether the treatment dose was repeated after vomiting were extracted as 'present' or 'absent'. Where a dose was repeated, the date and time of the repeated dose were used to calculate time from dosing.

1.1.5.2 Concomitant Medications

Concomitant medications were extracted as a list of drug names as recorded in the original data if present.

1.1.5.3 Antimalarial Pre-treatment

Antimalarial pre-treatment was extracted as 'present' or 'absent' with the name of the pre-treatment drug extracted as free text into a separate column where present.

1.1.5.4 Drug Concentrations

Amodiaquine and desethylamodiaquine concentrations in plasma were extracted as standardised to units of nmol/litre.

1.2 DATA INTEGRITY CHECKS

Individual patient data were checked for completeness, as well as for invalid, out-of-range, or inconsistent entries. Values incompatible with what would be observed in malaria clinical trials were considered missing. Queries were raised with study investigators and resolved where possible.

1.3 DATA ANALYSIS

1.3.1 Study-Specific Heart Rate Correction

The correction exponent is the coefficient of $logRR$ from the log-log linear regression:

$$logQT \sim logRR\ +\ sex\ +\ temperature\ + ECGday * drug +\ (1 \,|\, patient)$$

In addition to antimalarial drug, the malaria disease and demographic variables included are those previously identified to have independent effects on the QT interval in malaria in adults[36].

1.3.2 Pharmacokinetic Analysis

Figure I: Population Pharmacokinetic Structural Model of Amodiaquine and Desethylamodiaquine[35]

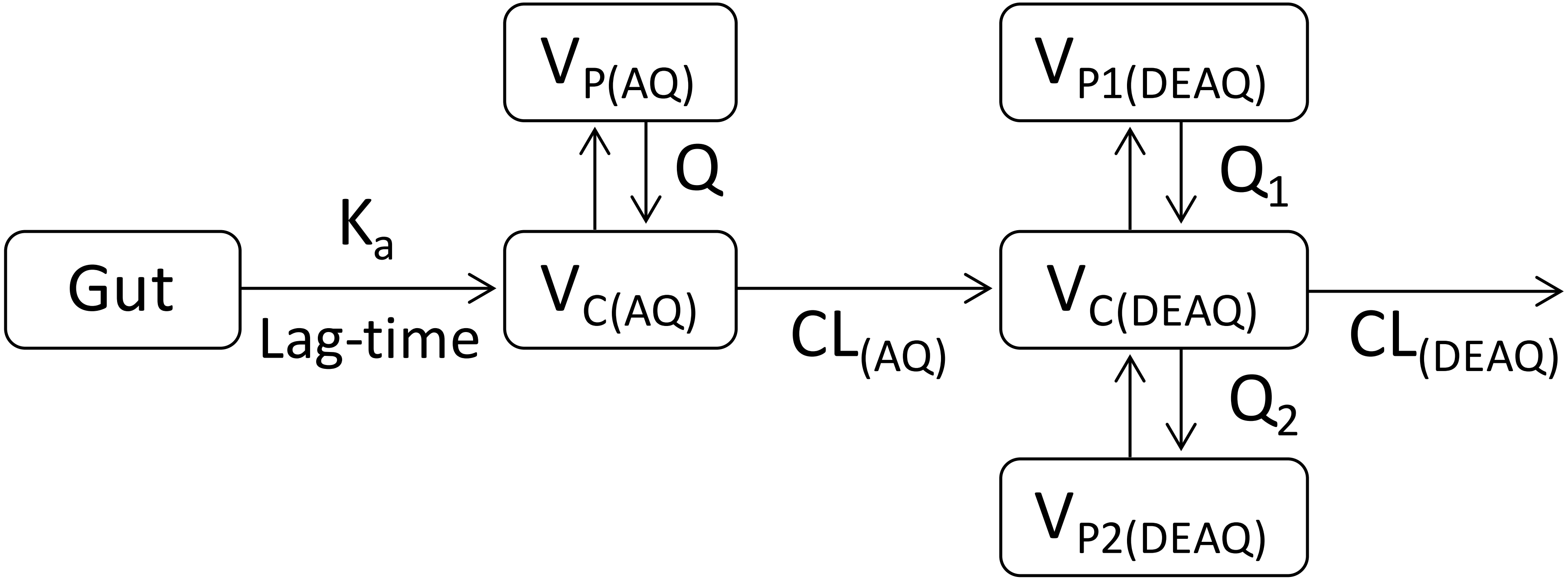

K_a = absorption first-order rate constant; $V_{C(AQ)}$ = apparent volume of distribution of the central amodiaquine compartment; $V_{P(AQ)}$ = apparent volume of distribution of the peripheral amodiaquine compartment; Q = inter-compartmental clearance rate of amodiaquine between its central and peripheral compartments; CL_{AQ} = apparent elimination clearance rate of amodiaquine from its central compartment to form desethylamodiaquine in the metabolite's central compartment; $V_{C(DEAQ)}$ = apparent volume of distribution of the central desethylamodiaquine compartment; $V_{P1(DEAQ)}$ = apparent volume of distribution of the first peripheral desethylamodiaquine compartment; $V_{P2(DEAQ)}$ = apparent volume of distribution of the second peripheral desthylamodiaquine compartment; Q_1 = inter-compartmental clearance rate of desethylamodiaquine between its central and first peripheral

compartments; Q_2 = inter-compartmental clearance rate of desethylamodiaquine between its central and second peripheral compartments; CL_{DEAQ} = apparent elimination clearance rate of desethylamodiaquine from its central compartment

1.3.2.1 Pharmacokinetic Modelling

Pharmacokinetic parameters were assumed to be log-normally distributed. Inter-individual variability was added to all parameters according to the following equation:

$$\theta_i = \theta_p \times \exp(\eta_{i,\theta})$$

where θ_i is the pharmacokinetic parameter estimate for the ith individual, θ_p is the population mean value of the investigated parameter, and $\eta_{i,\theta}$ is the deviation of the ith individual estimate from the population parameter value. Inter-individual variability was assumed to be normally distributed with mean zero and variance ω^2 (diagonal correlation matrix). Where estimates were <10% or had a relative standard error of >50%, inter-individual variability was fixed to zero. The residual unexplained variability in concentration was described by an additive error on the individually predicted logarithmic concentrations which is equivalent to an exponential error for non-transformed concentrations on the arithmetic scale.

Individual body weight, scaled by the median body weight (48.0kg) of the previous study[35] population, was included as a fixed allometric function to all clearance (power of 0.75) and volume of distribution (power of 1) parameters.

Model discrimination was based on the objective function value (OFV) which is proportional to -2 times the log likelihood of the data and has a Chi-squared distribution. A likelihood ratio test with a reduction in OFV of 3.84 or more was considered significant at $p = 0.05$ for a nested model with a difference of one degree of freedom. Goodness-of-fit plots were used to identify potential model misspecification and systematic errors. Model robustness and parameter confidence intervals were evaluated using a sampling-importance-resampling procedure[57]. Predictive performance was assessed with prediction-corrected visual and numerical predictive checks (n = 2000)[58].

1.3.3 Concentration-Effect Analyses

Variable Selection

Variable selection was based on directed acyclic graphs of proposed causal relationships among collected variables informed by literature review and expert consultation used to determine minimal sufficient adjustment sets for regression modelling.

Corrected QT Interval Models

QTcS ~ total drug concentration + Δtemperature + age + sex + ΔRR interval + (1|patient)

QTcF ~ total drug concentration + Δtemperature + age + sex + ΔRR interval + (1|patient)

QTcB ~ total drug concentration + Δtemperature + age + sex + ΔRR interval + (1|patient)

where $QTcS = \frac{QT}{RR^{0.42}}$ & $QTcF = \frac{QT}{\sqrt[3]{RR}}$ & $QTcB = \frac{QT}{\sqrt[2]{RR}}$, and RR is in units of seconds

QRS & PR Interval Models

QRS ~ total drug concentration + Δtemperature + age + sex + ΔRR interval + (1|patient)

PR ~ total drug concentration + Δtemperature + age + sex + ΔRR interval + (1|patient)

Change in Pulse Rate Model

ΔHR ~ total drug concentration + malaria + Δtemperature + sex + (1|patient)

Change in Blood Pressure Models

ΔSBP ~ total drug concentration + malaria + (1|patient) [supine]

ΔDBP ~ total drug concentration + malaria + (1|patient) [supine]

ΔSBPe ~ total drug concentration + malaria + (1|patient) [erect]

ΔDBPe ~ total drug concentration + malaria + (1|patient) [erect]

ΔpSBP ~ total drug concentration + malaria + (1|patient) [postural drop = supine - erect]

ΔpDBP ~ total drug concentration + malaria + (1|patient) [postural drop = supine - erect]

Figure II: Directed Acyclic Graph of Factors Affecting the Heart Rate in Malaria in Adults after Amodiaquine Treatment

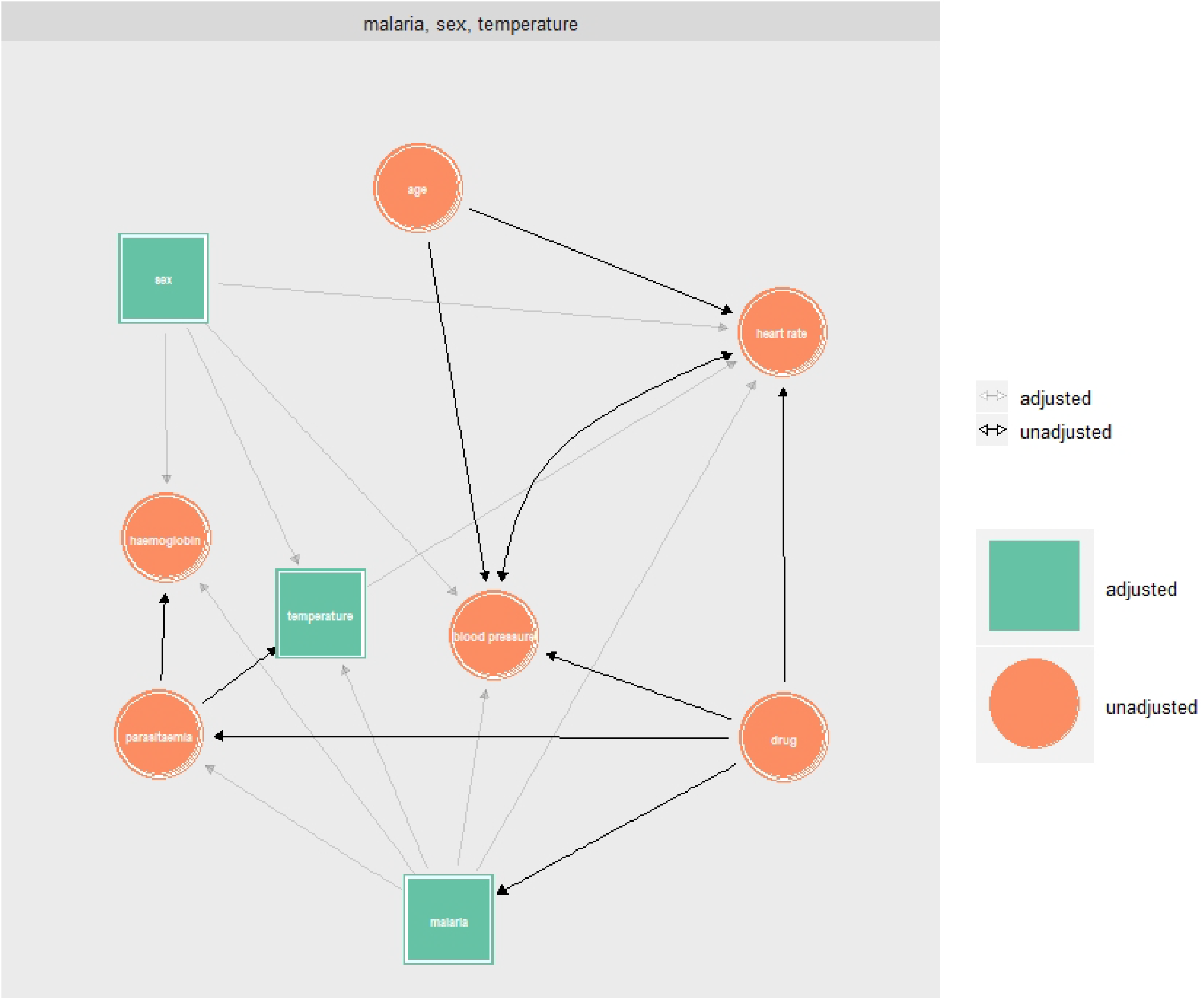

Directed acyclic graph generated in DAGitty[59] describing proposed causal relationships among factors affecting the heart rate in malaria in adults after antimalarial treatment with amodiaquine showing minimal sufficient covariate adjustment set (facet label & green squares). Bidirectional arrows do not represent reciprocal causation but depict unobserved confounders. The minimal adjustment set consisting of disease variables of malaria and temperature along with the demographic covariate of sex were included as fixed effects in multivariable linear mixed effects analyses.

Figure III: Directed Acyclic Graph of Factors Affecting Blood Pressure in Malaria After Amodiaquine Treatment

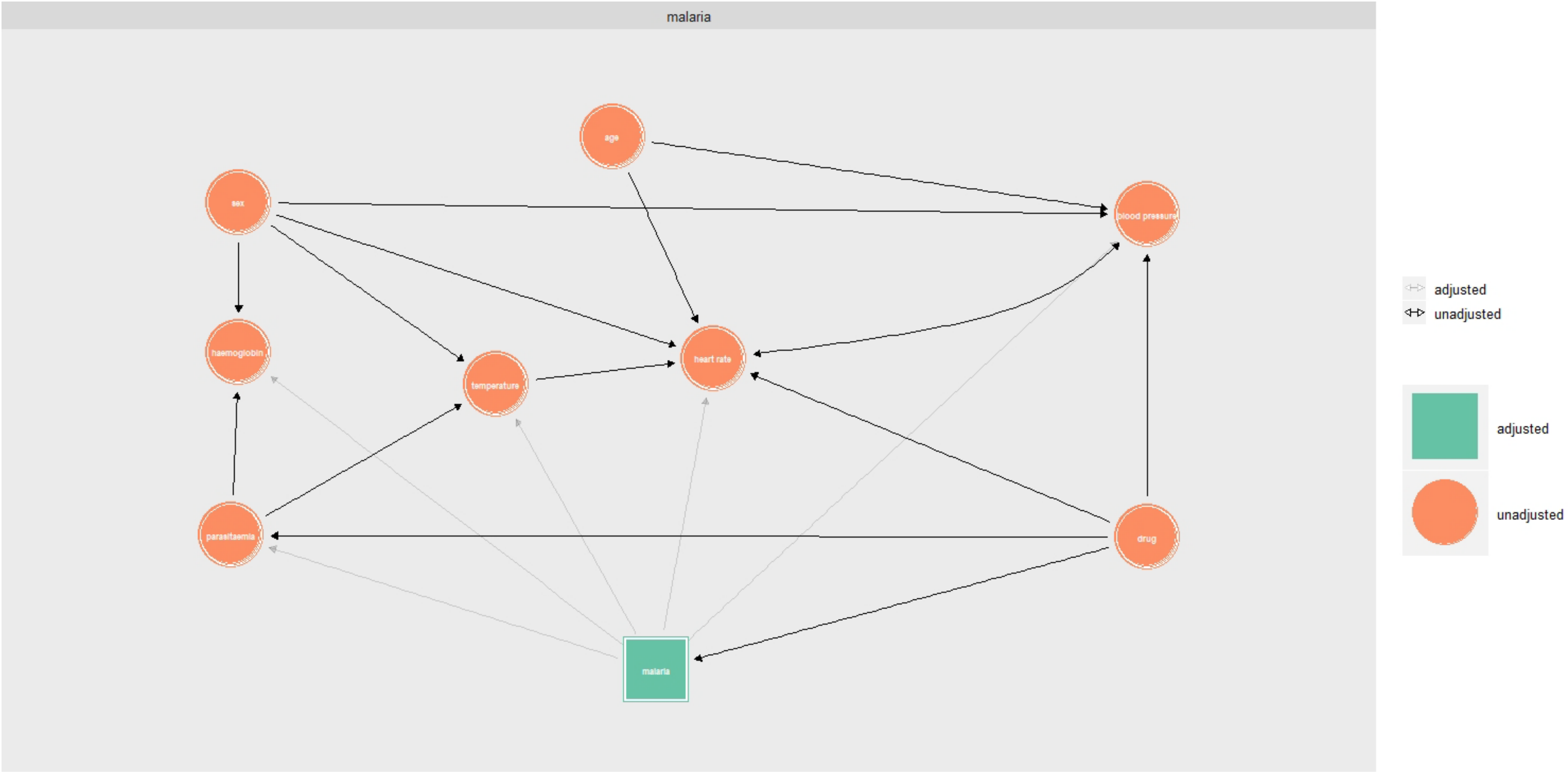

Directed acyclic graph generated in DAGitty[59] describing proposed causal relationships among factors affecting blood pressure in malaria after antimalarial treatment with amodiaquine showing minimal sufficient covariate adjustment set (facet label & green squares). The minimal adjustment set consisting of the malaria disease variable was included as a fixed effect in multivariable linear mixed effects analyses.

Figure IV: Directed Acyclic Graph of Factors Affecting the Electrocardiographic QT Interval in Malaria after Amodiaquine Treatment

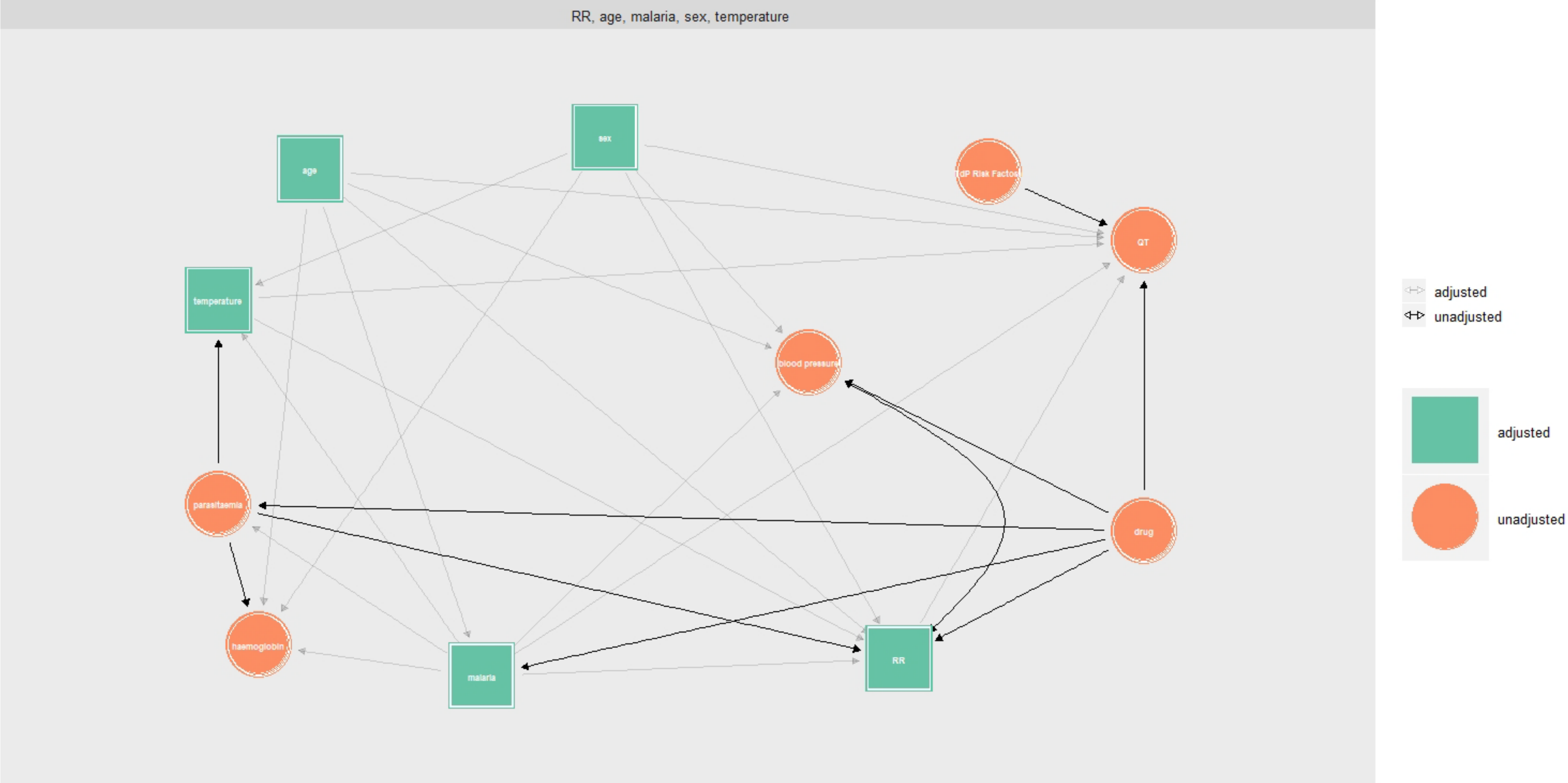

Directed acyclic graph generated in DAGitty[59] describing proposed causal relationships among factors affecting the electrocardiographic QT interval in malaria after antimalarial treatment with amodiaquine showing minimal sufficient covariate adjustment set (facet label & green squares). Bidirectional arrows do not represent reciprocal causation but depict unobserved confounders. The minimal adjustment set consisting of malaria disease variables of malaria and temperature along with demographic covariates of age and sex were included as fixed effects in multivariable linear mixed effects analyses. A study-specific heart rate correction factor was used for RR interval-related confounding.

Figure V: Directed Acyclic Graph of Factors Affecting the Electrocardiographic QRS & PR Intervals in Malaria after Amodiaquine Treatment

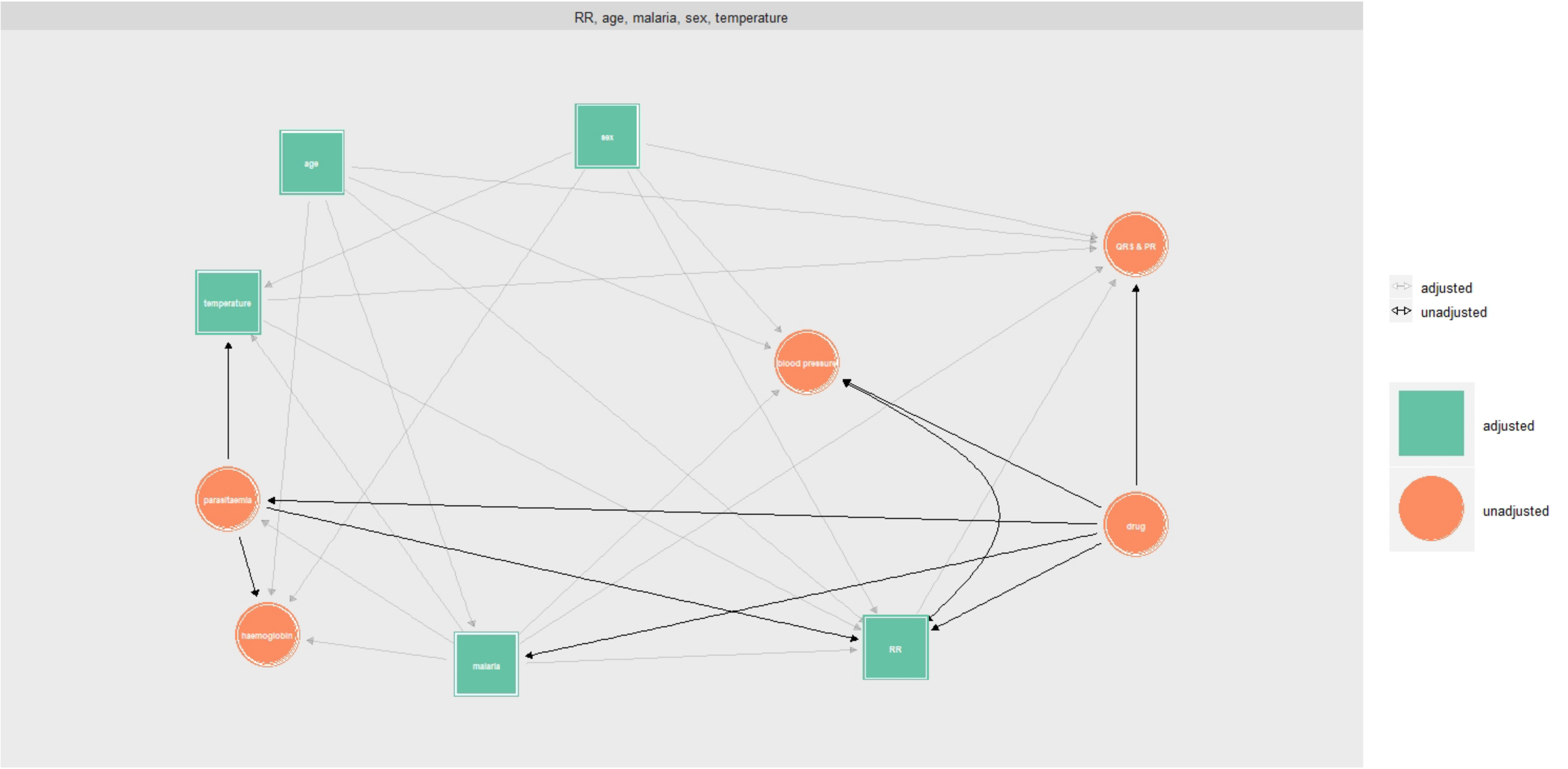

Directed acyclic graph generated in DAGitty[59] describing proposed causal relationships among factors affecting the electrocardiographic QRS and PR intervals in malaria after antimalarial treatment with amodiaquine showing minimal sufficient covariate adjustment set (facet label & green squares). Bidirectional arrows do not represent reciprocal causation but depict unobserved confounders. The minimal adjustment set consisting of malaria disease variables of malaria and temperature along with demographic covariates of age and sex were included as fixed effects in multivariable linear mixed effects analyses. A study-specific heart rate correction factor was used for RR interval-related confounding.

2 Supplementary Results

2.1 PHARMACOKINETIC ANALYSIS

Figure VI: Observed Plasma Concentrations of Amodiaquine and Desethylamodiaquine

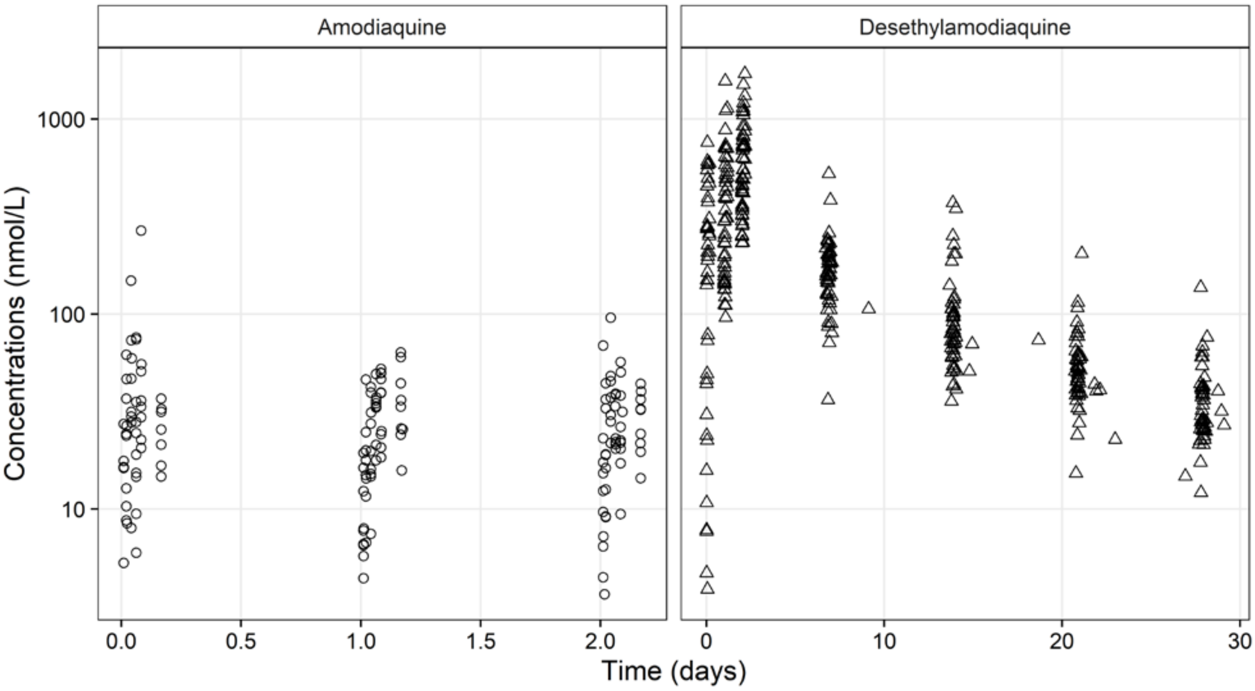

Observed plasma concentrations of amodiaquine (open circles) and desethylamodiaquine (open triangles) from sparse sampling

Table I: Population Pharmacokinetic Parameter Estimates

Population Pharmacokinetic Parameters	Prior Estimates*	Population Estimates†	95% Confidence Interval‡	%RSE‡
Amodiaquine				
K_a (hour^{-1})	0.418	0.469	0.410–0.508	5.45%
Absorption lag time (hour)	0.314	0.226	0.214–0.233	2.28%
$V_{C(AQ)}$ (litres)	3520	5840	4520–7110	11.1%
$CL_{(AQ)}$ (litres/hour)	2420	2790	2510–3070	4.80%
$V_{P(AQ)}$ (litres)	32500	34200	29600–40100	7.85%
$Q_{(AQ)}$ (litres/hour)	2470	2900	2400–3350	8.09%
σ_{AQ}	NA	0.108	0.0763–0.145	8.42%
Desethylamodiaquine				
$V_{C(DEAQ)}$ (litres)	198	213	158–272	13.6%
$CL_{(DEAQ)}$ (litres/hour)	34.1	35.2	33.3–37.8	3.25%
$V_{P1(DEAQ)}$ (litres)	2630	2760	2310–3180	7.79%
$Q_{P1(DEAQ)}$ (litres/hour)	163	181	157–206	13.6%
$V_{P2(DEAQ)}$ (litres)	5650	6530	5740–7410	6.24%
$Q_{P2(DEAQ)}$ (litres/hour)	25.4	30	25.6–35.2	7.98%
σ_{DEAQ}	NA	0.255	0.216–0.304	4.49%
Inter-Individual Variability (%CV)				
Absorption lag time (hour)	0.359 (65.7%)	0.541 (73.6%)	0.342–0.738	9.03%
$V_{C(AQ)}$ (litres)	0.382 (68.2%)	0.772 (87.9%)	0.383–1.34	16.1%
$CL_{(AQ)}$ (litres/hour)	0.0578 (24.4%)	0.0586 (24.2%)	0.0368–0.0890	12.5%
$V_{P(AQ)}$ (litres)	0.0603 (24.9%)	0.0612 (27.4%)	0.0337–0.0814	10.0%
$Q_{(AQ)}$ (litres/hour)	0.0678 (26.5%)	2.94 (171%)	1.95–4.56	11.4%
$V_{C(DEAQ)}$ (litres)	0.196 (46.5%)	0.691 (83.1%)	0.335–1.08	13.4%
$CL_{(DEAQ)}$ (litres/hour)	0.0522 (23.1%)	0.0373 (19.3%)	0.0255–0.0532	9.73%
$V_{P2(DEAQ)}$ (litres)	0.0239 (15.6%)	0.0211 (14.5%)	0.0132–0.0298	9.87%
F	0.0251 (15.9%)	0.0256 (16.0%)	0.0164–0.0385	10.8%
Secondary Parameters				
$C_{max(AQ)}$ (ng/ml)		13.8	7.86-30.6	
$C_{max(AQ)}$ (nmol/litre)		42.1	24.0-93.3	
$T_{max(AQ)}$ (hours)		1.70	0.793-3.56	
$t_{1/2(AQ)}$ (hours)		5.93	0.861-72.1	
$AUC_{7\ days}$ (hours x ng/ml)		494	341-681	
$C_{max(DEAQ)}$ (ng/ml)		240	166-429	
$C_{max(DEAQ)}$ (nmol/litre)		731	506-1310	
$T_{max(DEAQ)}$ (hours)		3.61	2.00-6.67	
$t_{1/2(DEAQ)}$ (days)		13.4	11.8-15.0	
$AUC_{28\ days}$ (hours x µg/ml)		31.7	19.8-46.3	

AQ = amodiaquine; DEAQ = desethylamodiaquine; K_a = absorption first-order rate constant; V_C = apparent volume of distribution of central compartment; CL = apparent elimination clearance rate from central compartment; V_P = apparent volume of distribution of peripheral compartment; Q = inter-compartmental clearance rate between central and peripheral compartment(s); σ = residual error variance; F = relative bioavailability; %CV = coefficient of variation for inter-individual variability computed as $100 \times \sqrt{\exp(\omega^2) - 1}$; C_{max} = maximum concentration; T_{max} = time to maximum concentration; $t_{1/2}$ = terminal elimination half-life; AUC = area under curve, i.e. total drug exposure; *Population pharmacokinetic estimates with corresponding parameter uncertainties from model developed from a separate study with rich pharmacokinetic sampling[35]; †Population mean estimates from NONMEM® for a 'typical' 48.0kg adult patient with uncomplicated malaria; ‡From sampling-importance-resampling procedure[57] of the final model.

Figure VII: Goodness-of-Fit Plots for the Final Pharmacokinetic Model

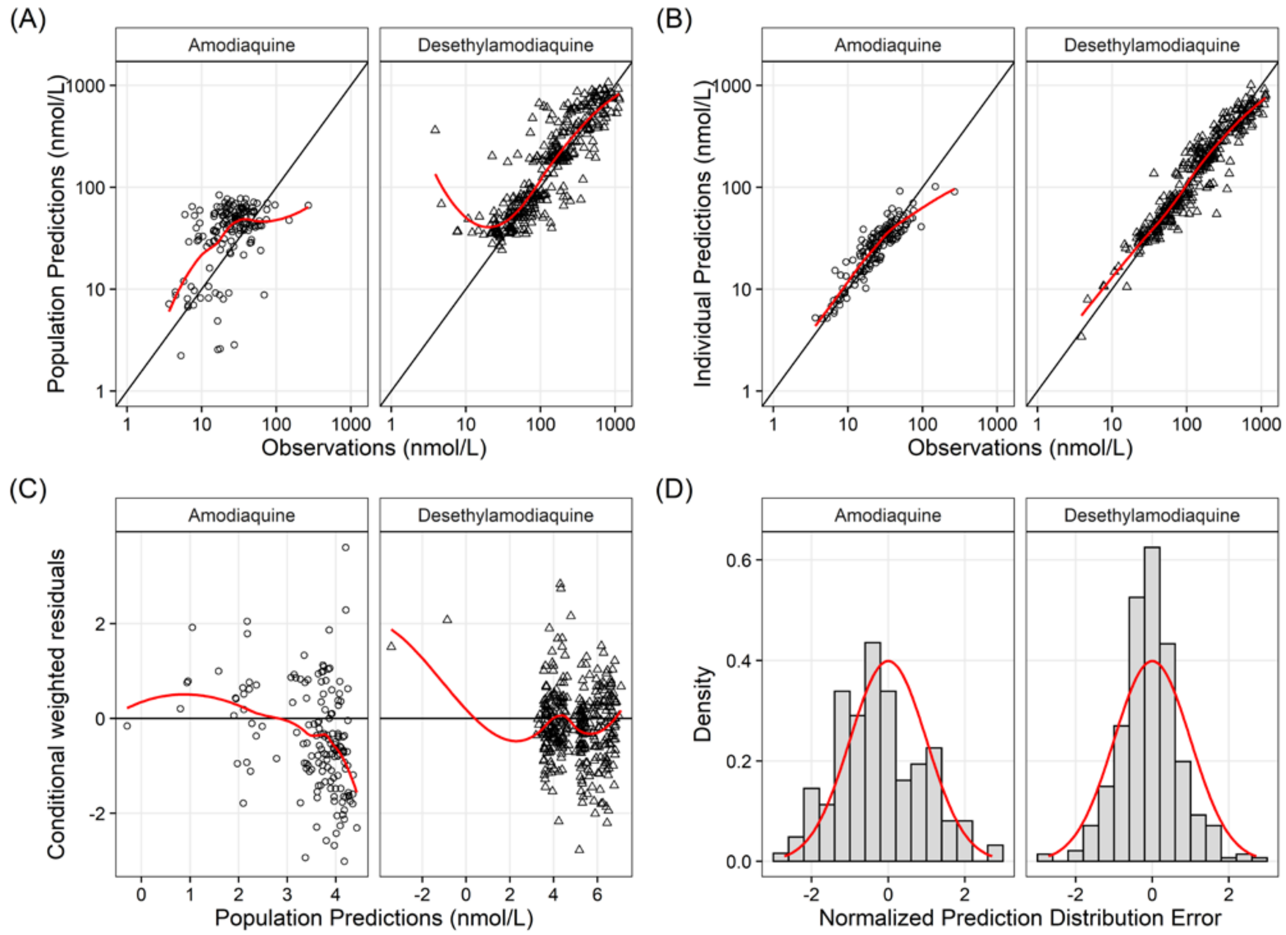

(A) Population predicted plasma concentrations versus observations, (B) Individual predicted concentrations versus observations, (C) Conditional weighted residuals versus population predicted concentrations, (D) Normalised prediction distribution error. In (A), (B), and (C), the solid black line is the identity line, and the solid red line is the locally weighted least squares regression line.

Figure VIII: Individual Observed and Predicted Concentrations over Time

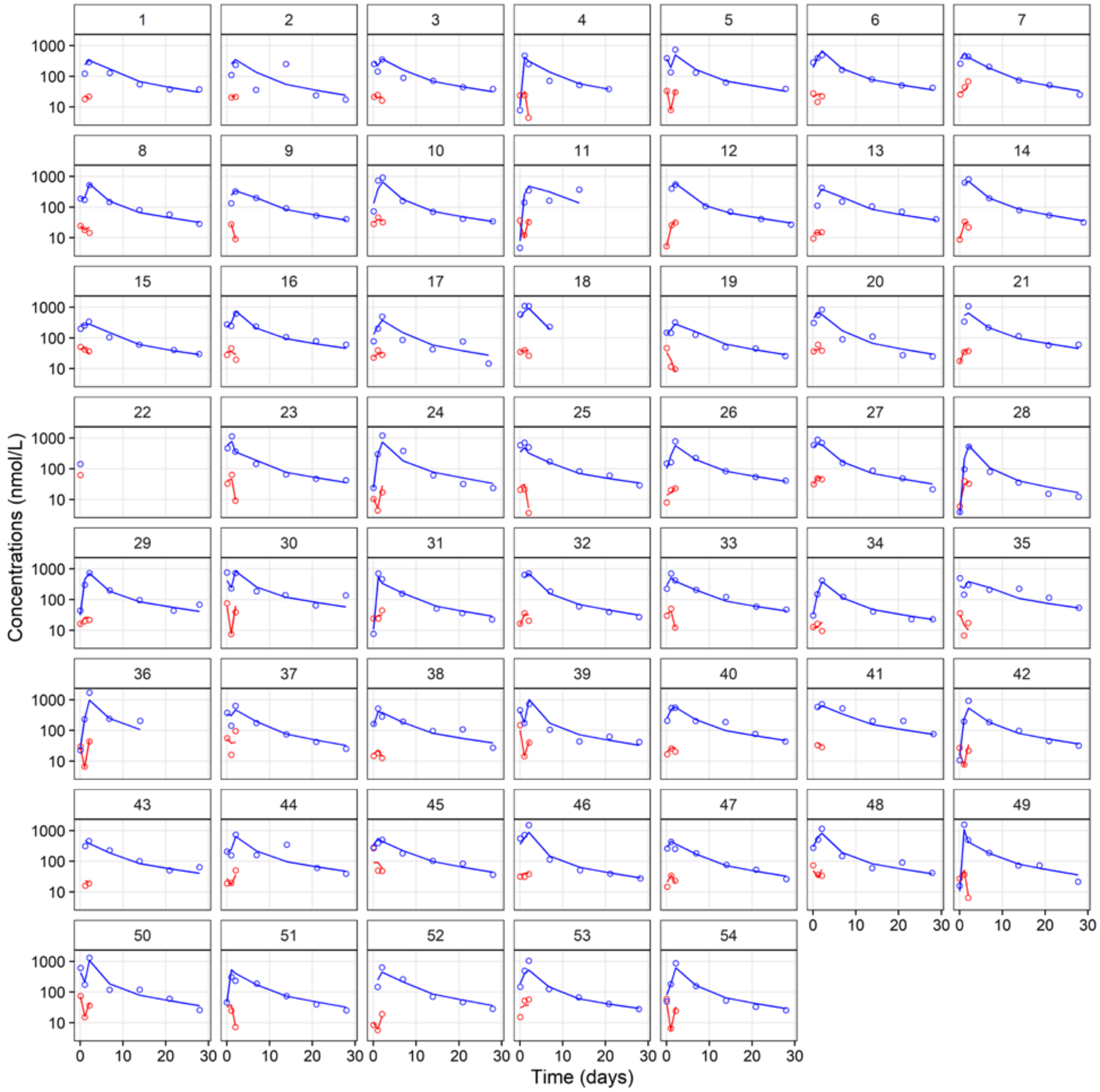

Observed (open circles) and predicted (solid lines) plasma concentrations for amodiaquine (red) and desthyl-amodiaquine (blue) over time for each individual patient (facet label)

Figure IX: Prediction-Corrected Visual Predictive Check for the Final Pharmacokinetic Model

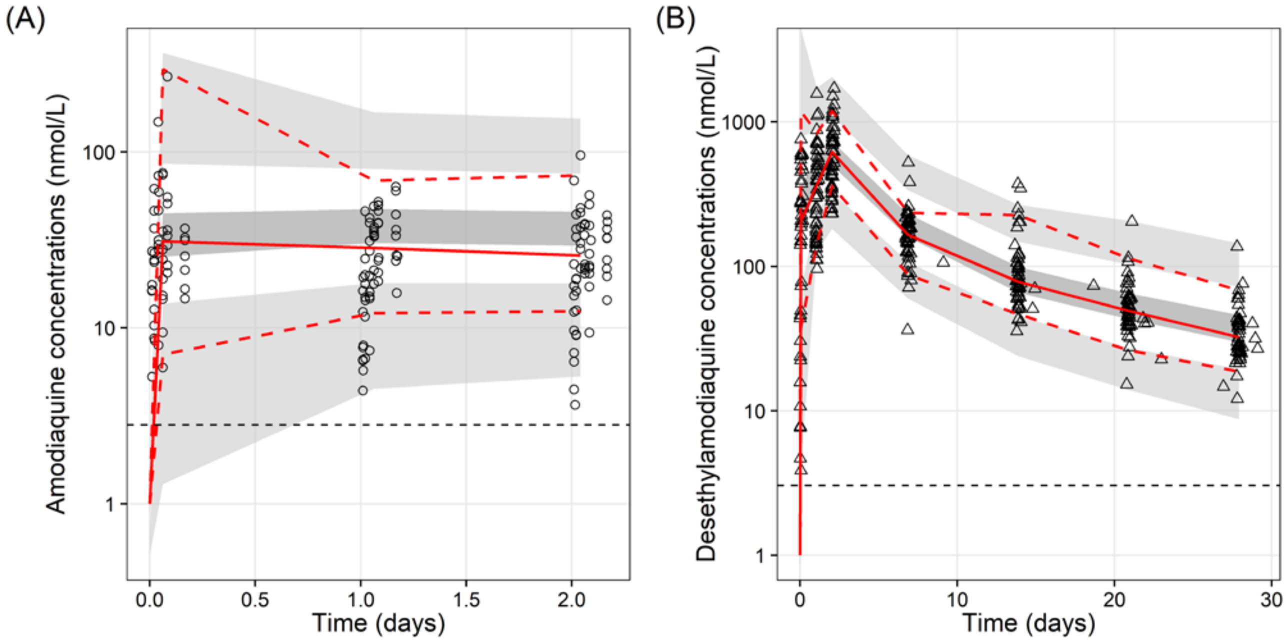

Visual predictive checks for the final pharmacokinetic model of (A) amodiaquine (open circles) and (B) desethylamodiaquine (open triangles) based on 2,000 stochastic simulations. Solid lines represent the median/50th percentile, while dashed lines represent the 5th and 95th percentiles of observed plasma concentrations. Shaded areas represent the 95% confidence intervals around the simulated 5th, 50th, and 95th percentiles.

Concentration-Effect Analyses

Table II: Factors Affecting Systolic Blood Pressure Parameters in Malaria Following Treatment with Amodiaquine

		Systolic Blood Pressure – Erect (mmHg)				*Systolic Blood Pressure – Postural Change (mmHg)*			
		**Univariable Analyses**		**Multivariable Analyses**		**Univariable Analyses**		**Multivariable Analyses**	
	**Number of Observations**	**Crude Estimate (95% CI)**	***p* value**	**Adjusted Estimate (95% CI)**	***p* value**	**Crude Estimate (95% CI)**	***p* value**	**Adjusted Estimate (95% CI)**	***p* value**
Total plasma concentration of amodiaquine and desethylamodiaquine, per 750* nmol/l increase	362	-12.33 (-15.21 to -9.46)	<0.0001	-11.05 (-14.75 to -7.35)	<0.0001	-5.01 (-8.06 to -1.97)	0.0013	-0.38 (-4.32 to 3.55)	0.849
Body temperature change, per 1°C increase	362	3.00 (1.76 to 4.24)	<0.0001			0.52 (-0.43 to 1.47)	0.2787		
Malaria	362								
Yes (days 0, 1, 2)	106	-4.73 (-6.91 to -2.55)	<0.0001	-0.39 (-2.92 to 2.14)	0.7617	-4.50 (-6.77 to -2.23)	0.0001	-4.35 (-7.10 to -1.59)	0.0021
No (days 3, 7, 14, 21, 28)	256	Reference		Reference		Reference		Reference	

*Mean maximum total plasma drug concentration (rounded) after a 3-day course of amodiaquine from pharmacokinetic analysis of same study

Table III: Factors Affecting Diastolic Blood Pressure Parameters in Malaria Following Treatment with Amodiaquine

		Diastolic Blood Pressure – Supine (mmHg)				*Diastolic Blood Pressure – Postural Change (mmHg)*			
		Univariable Analyses		Multivariable Analyses		Univariable Analyses		Multivariable Analyses	
	Number of Observations	Crude Estimate (95% CI)	p value	Adjusted Estimate (95% CI)	p value	Crude Estimate (95% CI)	p value	Adjusted Estimate (95% CI)	p value
Total plasma concentration of amodiaquine and desethylamodiaquine, per 750* nmol/l increase	362	-7.97 (-10.25 to -5.69)	<0.0001	-4.65 (-7.37 to -1.94)	0.0008	2.31 (-0.10 to 4.71)	0.0602	4.87 (1.90 to 7.84)	0.0014
Body temperature change, per 1°C increase	362	2.08 (1.11 to 3.05)	<0.0001			-0.50 (-1.14 to 0.15)	0.1331		
Malaria	362								
Yes (days 0, 1, 2)	106	-2.17 (-3.71 to -0.63)	0.0058	-0.35 (-2.20 to 1.51)	0.7141	-1.00 (-2.75 to 0.75)	0.2621	-2.94 (-5.03 to -0.85)	0.0059
No (days 3, 7, 14, 21, 28)	256	Reference		Reference		Reference		Reference	

*Mean maximum total plasma drug concentration (rounded) after a 3-day course of amodiaquine from pharmacokinetic analysis of same study

Table IV: Factors Affecting the Electrocardiogram QRS and PR Intervals in Malaria Following Treatment with Amodiaquine

		QRS Interval (milliseconds)				*PR Interval (milliseconds)*			
		Univariable Analyses		Multivariable Analyses		Univariable Analyses		Multivariable Analyses	
	Number of Observations	Crude Estimate (95% CI)	p value	Adjusted Estimate (95% CI)	p value	Crude Estimate (95% CI)	p value	Adjusted Estimate (95% CI)	p value
Total plasma concentration of amodiaquine and desethylamodiaquine, per 750* nmol/l increase	356	2.34 (0.38 to 4.29)	0.0194	-0.47 (-2.51 to 1.57)	0.6525	6.68 (3.51 to 9.84)	<0.0001	2.01 (-1.29 to 5.31)	0.2313
Body temperature change, per 1°C increase	356	-2.08 (-2.86 to -1.30)	<0.0001	-0.30 (-1.42 to 0.82)	0.5953	-3.86 (-5.15 to -2.57)	<0.0001	-0.54 (-2.38 to 1.30)	0.5647
Sex	356								
Female	187	Reference		Reference		Reference		Reference	
Male	169	5.44 (0.68 to 10.2)	0.0259	5.09 (0.27 to 9.91)	0.0388	1.60 (-11.84 to 15.04)	0.8124	-0.06 (-13.72 to 13.62)	0.9935
Age, per 10-year increase	356	1.53 (-0.76 to 3.82)	0.186	1.08 (-1.17 to 3.34)	0.3386	4.03 (-2.17 to 10.22)	0.1976	4.11 (-2.29 to 10.51)	0.2027
RR interval change, per 300 millisecond increase	356	4.43 (3.15 to 5.71)	<0.0001	4.20 (2.22 to 6.18)	<0.0001	8.18 (6.10 to 10.26)	<0.0001	6.91 (3.68 to 10.14)	<0.0001

*Mean maximum total plasma drug concentration (rounded) after a 3-day course of amodiaquine from pharmacokinetic analysis of same study

†Mean change in RR interval from baseline (rounded) after last dose of amodiaquine treatment in this study

3 References

1. Wiselogle FY. A Survey of Antimalarial Drugs 1941-1945. Ann Arbor, Michigan: J. W. Edwards; 1946.
2. Burckhalter JH, Tendick FH, et al. Aminoalkylphenols as antimalarials (heterocyclicamino)-alpha-amino-o-cresols; the synthesis of camoquin. *J Am Chem Soc* 1948; **70**(4): 1363-73.
3. World Health Organization. Guidelines for the Treatment of Malaria. 3rd ed. Geneva, Switzerland; 2015.
4. World Health Organization. World Malaria Report 2019. Geneva, Switzerland, 2019.
5. World Health Organization. Seasonal malaria chemoprevention with sulfadoxine-pyrimethamine plus amodiaquine in children: A field guide. Geneva, Switzerland, 2013.
6. Arora RB, Madan BR, Pathak RK. Antiarrhythmics. VIII. Chloroquine, amodiaquine, procaine amide and quinidine in experimental auricular arrhythmias simulating clinical disorders. *Indian J Med Res* 1956; **44**(3): 453-62.
7. Arora RB, Madan BR. Antiarrhythmics. II. Amodiaquin (camoquin) in cardiac arrhythmias. *Indian J Med Res* 1956; **44**(1): 99-106.
8. Arora RB, Lal A. Antimalarial Drugs on the Automaticity of Sino-Auricular and Atrio-Ventricular Nodes. *Indian J Med Res* 1963; **51**: 725-32.
9. Chan XHS, Haeusler IL, Win YN, et al. The cardiovascular effects of amodiaquine and structurally related antimalarials: An individual patient data meta-analysis. *PLoS Med* 2021; **18**(9): e1003766.
10. Assi SB, Aba YT, Yavo JC, et al. Safety of a fixed-dose combination of artesunate and amodiaquine for the treatment of uncomplicated Plasmodium falciparum malaria in real-life conditions of use in Cote d'Ivoire. *Malar J* 2017; **16**(1): 8.
11. Dodoo AN, Fogg C, Nartey ET, et al. Profile of adverse events in patients receiving treatment for malaria in urban Ghana: a cohort-event monitoring study. *Drug Saf* 2014; **37**(6): 433-48.
12. Bassi PU, Osakwe AI, Isah A, et al. Safety of Artemisinin-Based Combination Therapies in Nigeria: A Cohort Event Monitoring Study. *Drug Saf* 2013; **36**(9): 747-56.
13. Whittaker DG, Capel RA, Hendrix M, et al. Cardiac TdP risk stratification modelling of anti-infective compounds including chloroquine and hydroxychloroquine. *R Soc Open Sci* 2021; **8**(4): 210235.
14. Capel RA, Herring N, Kalla M, et al. Hydroxychloroquine reduces heart rate by modulating the hyperpolarization-activated current If: Novel electrophysiological insights and therapeutic potential. *Heart Rhythm* 2015; **12**(10): 2186-94.
15. Borsini F, Crumb W, Pace S, et al. In vitro cardiovascular effects of dihydroartemisinin-piperaquine combination compared with other antimalarials. *Antimicrob Agents Chemother* 2012; **56**(6): 3261-70.
16. Noujaim SF, Stuckey JA, Ponce-Balbuena D, et al. Structural bases for the different anti-fibrillatory effects of chloroquine and quinidine. *Cardiovasc Res* 2011; **89**(4): 862-9.
17. Traebert M, Dumotier B, Meister L, Hoffmann P, Dominguez-Estevez M, Suter W. Inhibition of hERG K+ currents by antimalarial drugs in stably transfected HEK293 cells. *Eur J Pharmacol* 2004; **484**(1): 41-8.
18. Adaramoye OA, Almeida MM. Vasorelaxation induced by amodiaquine in rat superior mesenteric arteries: in vivo and in vitro studies. *Acta Pol Pharm* 2010; **67**(5): 529-36.
19. Lim LY, Go ML. The anticholinesterase activity of mefloquine. *Clin Exp Pharmacol Physiol* 1985; **12**(5): 527-31.
20. Go ML, Ngiam TL, Wan AS. Investigation of the anti-acetylcholinesterase activities of the antimalarial agent, amodiaquine, and related compounds. *Southeast Asian J Trop Med Public Health* 1981; **12**(1): 37-41.

21. Churchill FC, Patchen LC, Campbell CC, Schwartz IK, Nguyen-Dinh P, Dickinson CM. Amodiaquine as a prodrug: importance of metabolite(s) in the antimalarial effect of amodiaquine in humans. *Life Sci* 1985; **36**(1): 53-62.
22. Hatton CS, Peto TE, Bunch C, et al. Frequency of severe neutropenia associated with amodiaquine prophylaxis against malaria. *Lancet* 1986; **1**(8478): 411-4.
23. Neftel KA, Woodtly W, Schmid M, Frick PG, Fehr J. Amodiaquine induced agranulocytosis and liver damage. *Br Med J (Clin Res Ed)* 1986; **292**(6522): 721-3.
24. Phillips-Howard PA, West LJ. Serious adverse drug reactions to pyrimethamine-sulphadoxine, pyrimethamine-dapsone and to amodiaquine in Britain. *J R Soc Med* 1990; **83**(2): 82-5.
25. World Health Organization. Practical Chemotherapy of Malaria. Report of a WHO Scientific Group. Geneva, Switzerland: World Health Organization, 1990.
26. Ogutu B, Juma E, Obonyo C, et al. Fixed dose artesunate amodiaquine - a phase IIb, randomized comparative trial with non-fixed artesunate amodiaquine. *Malar J* 2014; **13**: 498.
27. Jullien V, Ogutu B, Juma E, Carn G, Obonyo C, Kiechel JR. Population pharmacokinetics and pharmacodynamic considerations of amodiaquine and desethylamodiaquine in Kenyan adults with uncomplicated malaria receiving artesunate-amodiaquine combination therapy. *Antimicrob Agents Chemother* 2010; **54**(6): 2611-7.
28. Ali AM, Penny MA, Smith TA, et al. Population Pharmacokinetics of the Antimalarial Amodiaquine: a Pooled Analysis To Optimize Dosing. *Antimicrob Agents Chemother* 2018; **62**(10).
29. Maude RJ, Plewes K, Faiz MA, et al. Does artesunate prolong the electrocardiograph QT interval in patients with severe malaria? *Am J Trop Med Hyg* 2009; **80**(1): 126-32.
30. R Core Team. R: A language and environment for statistical computing. Vienna, Austria: R Foundation for Statistical Computing; 2018.
31. Pinheiro J, Bates D, DebRoy S, Sarkar D, R Core Team. nlme: Linear and Nonlinear Mixed Effects Models. R package version 3.1-141 ed; 2019.
32. Bauer RJ. NONMEM Users Guide: Introduction to NONMEM 7.4.1. Gaithersburg, Maryland: ICON plc; 2017.
33. Keizer R, van Benten M, Beijnen J, Schellens J, Huitema A. Pirana and PCluster: a modeling environment and cluster infrastructure for NONMEM. *Computer Methods and Programs in Biomedicine* 2011; **101**(1): 72-9.
34. Lindbom L, Ribbing J, Jonsson E. Perl-speaks-NONMEM (PsN)--a Perl module for NONMEM related programming. *Computer Methods and Programs in Biomedicine* 2004; **75**(2): 85-94.
35. Tarning J, Chotsiri P, Jullien V, et al. Population pharmacokinetic and pharmacodynamic modeling of amodiaquine and desethylamodiaquine in women with Plasmodium vivax malaria during and after pregnancy. *Antimicrob Agents Chemother* 2012; **56**(11): 5764-73.
36. Chan XHS, Win YN, Haeusler IL, et al. Factors affecting the electrocardiographic QT interval in malaria: A systematic review and meta-analysis of individual patient data. *PLoS Med* 2020; **17**(3): e1003040.
37. World Health Organization. WHO Evidence Review Group on the Cardiotoxicity of Antimalarial Medicines. Geneva, Switzerland, 2017.
38. Adjei GO, Oduro-Boatey C, Rodrigues OP, et al. Electrocardiographic study in Ghanaian children with uncomplicated malaria, treated with artesunate-amodiaquine or artemether-lumefantrine. *Malar J* 2012; **11**: 420.
39. Li XQ, Bjorkman A, Andersson TB, Ridderstrom M, Masimirembwa CM. Amodiaquine clearance and its metabolism to N-desethylamodiaquine is mediated by CYP2C8: a new high affinity and turnover enzyme-specific probe substrate. *J Pharmacol Exp Ther* 2002; **300**(2): 399-407.
40. European Medicines Agency. Artesunate Amodiaquine Winthrop Tablets: Summary of Product Characteristics. 2014.
41. Haeusler IL, Chan XHS, Guerin PJ, White NJ. The arrhythmogenic cardiotoxicity of the quinoline and structurally related antimalarial drugs: a systematic review. *BMC Med* 2018; **16**(1): 200.

42. ter Kuile FO, Nosten F, Luxemburger C, et al. Mefloquine treatment of acute falciparum malaria: a prospective study of non-serious adverse effects in 3673 patients. *Bull World Health Organ* 1995; **73**(5): 631-42.
43. Costa S, Saguner AM, Gasperetti A, Akdis D, Brunckhorst C, Duru F. The Link Between Sex Hormones and Susceptibility to Cardiac Arrhythmias: From Molecular Basis to Clinical Implications. *Front Cardiovasc Med* 2021; **8**: 644279.
44. Strahan JH. Quinine by continuous intravenous drip in the treatment of acute falciparum malaria. *Trans R Soc Trop Med Hyg* 1948; **41**(5): 669-76.
45. White NJ, Miller KD, Churchill FC, et al. Chloroquine treatment of severe malaria in children. Pharmacokinetics, toxicity, and new dosage recommendations. *N Engl J Med* 1988; **319**(23): 1493-500.
46. White NJ. Cardiotoxicity of antimalarial drugs. *Lancet Infect Dis* 2007; **7**(8): 549-58.
47. Supanaranond W, Davis TM, Pukrittayakamee S, Nagachinta B, White NJ. Abnormal circulatory control in falciparum malaria: the effects of antimalarial drugs. *Eur J Clin Pharmacol* 1993; **44**(4): 325-9.
48. ICH Harmonised Tripartite Guideline E14. The Clinical Evaluation of QT/QTc Interval Prolongation and Proarrhythmic Potential for Non-Antiarrhythmic Drugs, 2005.
49. Chan XHS, Win YN, Mawer LJ, Tan JY, Brugada J, White NJ. Risk of sudden unexplained death after use of dihydroartemisinin-piperaquine for malaria: a systematic review and Bayesian meta-analysis. *Lancet Infect Dis* 2018; **18**(8): 913-23.
50. Rabkin SW, Szefer E, Thompson DJS. A New QT Interval Correction Formulae to Adjust for Increases in Heart Rate. *JACC Clin Electrophysiol* 2017; **3**(7): 756-66.
51. Wattanakul T, Ogutu B, Kabanywanyi AM, et al. Pooled Multicenter Analysis of Cardiovascular Safety and Population Pharmacokinetic Properties of Piperaquine in African Patients with Uncomplicated Falciparum Malaria. *Antimicrob Agents Chemother* 2020; **64**(7).
52. Chotsiri P, Wattanakul T, Hoglund RM, et al. Population pharmacokinetics and electrocardiographic effects of dihydroartemisinin-piperaquine in healthy volunteers. *Br J Clin Pharmacol* 2017; **83**(12): 2752-66.
53. Chotsiri P, Tarning J, Hoglund RM, Watson J, White NJ. Pharmacometric and electrocardiographic evaluation of chloroquine and azithromycin in healthy volunteers. *Clin Pharmacol Ther* 2022.
54. Funck-Brentano C, Ouologuem N, Duparc S, et al. Evaluation of the effects on the QT-interval of 4 artemisinin-based combination therapies with a correction-free and heart rate-free method. *Sci Rep* 2019; **9**(1): 883.
55. Ngouesse B, Basco LK, Ringwald P, Keundjian A, Blackett KN. Cardiac effects of amodiaquine and sulfadoxine-pyrimethamine in malaria-infected African patients. *Am J Trop Med Hyg* 2001; **65**(6): 711-6.
56. Chue AL, Moore RL, Cavey A, et al. Comparability of tympanic and oral mercury thermometers at high ambient temperatures. *BMC Res Notes* 2012; **5**: 356.
57. Dosne AG, Bergstrand M, Karlsson MO. An automated sampling importance resampling procedure for estimating parameter uncertainty. *J Pharmacokinet Pharmacodyn* 2017; **44**(6): 509-20.
58. Bergstrand M, Hooker AC, Wallin JE, Karlsson MO. Prediction-corrected visual predictive checks for diagnosing nonlinear mixed-effects models. *AAPS J* 2011; **13**(2): 143-51.
59. Textor J, van der Zander B, Gilthorpe MS, Liskiewicz M, Ellison GT. Robust causal inference using directed acyclic graphs: the R package 'dagitty'. *Int J Epidemiol* 2016; **45**(6): 1887-94.
